# Synthesis of Novel 3-Deoxy-3-thio Derivatives of d-Glucosamine and Their Application as Ligands for the Enantioselective Addition of Diethylzinc to Benzaldehyde

**DOI:** 10.3390/ijms25105542

**Published:** 2024-05-19

**Authors:** Yusuf Zaim Hakim, Tomasz Bauer

**Affiliations:** Faculty of Chemistry, University of Warsaw, L. Pasteura 1, 02-093 Warsaw, Poland; y.hakim@student.uw.edu.pl

**Keywords:** benzaldehyde, enantioselectivity, d-glucosamine, diethylzinc, thiol

## Abstract

A series of novel thio-derivatives of d-glucosamine has been synthesized using double inversion procedures at the C3 atom. New compounds were applied as ligands for the diethylzinc addition to benzaldehyde and the products of the addition were obtained with a low to good enantiomeric ratio. The direction and the level of the asymmetric induction were highly dependent on the type of protecting groups on the nitrogen and sulfur atoms.

## 1. Introduction

The asymmetric addition of dialkylzinc compounds to aldehydes is an important method for the synthesis of optically active secondary alcohols. Due to the low reactivity of dialkylzinc reagents toward the carbonyl group, the addition requires the presence of a catalyst, usually an amino alcohol, which increases the rate of reaction and often controls the stereochemical outcome of the alcohol product. Since the first efficient chiral catalyst, (-)-3-exo (dimethylamino)isoborneol (DAIB), was introduced by Noyori [1], syntheses and applications of several efficient chiral catalysts have been reported [2,3].

**Figure 1:**
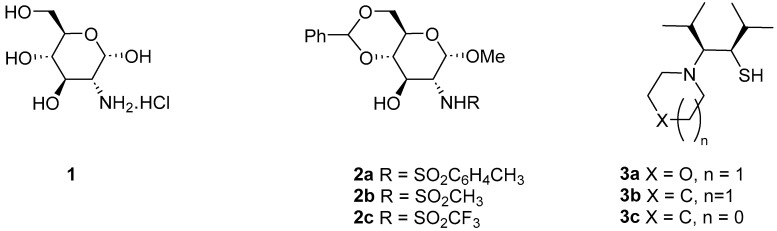
Ligands for the enantioselective additions of organozinc compounds.

Derivatives of common, simple carbohydrates are not frequently reported among the many different forms of chiral ligands that are accessible. The benefit of sugar derivatives is their modular synthesis, which allows for easy ligand structure modification throughout synthesis, resulting in different ligands possessing the same chiral precursor. We previously reported the synthesis of hydroxy sulfonamides **2** derived from d-glucosamine **1**, which were used as ligands in the titanium-promoted additions of diethyl- [4,5], and alkynylzincs [6] to aldehydes (Figure 1). 

It has been observed that β-amino thiol can be viable alternative to β-amino alcohol as a catalyst for asymmetric organozinc addition because of certain crucial features, including the diminished tendency of metal thiolates to reduce the Lewis acidity of the metal in comparison to metal alcoholates, higher affinity of thiols to zinc and increased polarizability of sulfur in comparison to the oxygen atom [7]. 

Several amino thiol ligands were reported, and one of the most efficient was the l-valine-derived amino thiols **3** developed by Tseng and Yang [8]. We have recently proved the efficacy of **3a** in the highly enantioselective alkenylation of aldehydes [9].

Herein, we present our studies on the synthesis of d-glucosamine derivatives bearing thiol functionality at the C3 position (sugar nomenclature) and their application for the diethylzinc addition to aldehydes.

The enantioselective addition of organozinc compounds is usually catalyzed by two types of ligands, namely β-hydroxy amines, as described by Noyori, and β-hydroxy sulfonamides or diols in the presence of tetraisopropoxytitanium, according to the mechanism described by Gau [10] and Walsh [11]. We decided to use our experience with the titanium-promoted addition of organozinc compounds in the presence of β-hydroxy sulfonamides **2** and investigate the respective thio-analogs. Additionally, as we expected the incompatibility of the hard Ti(O*i*Pr)_4_ with the soft sulfur atom, we planned a synthesis of the thio-analogs of the classical β-hydroxy amines earlier described by Davis and co-workers [12].

## 2. Results

**Scheme 1:**
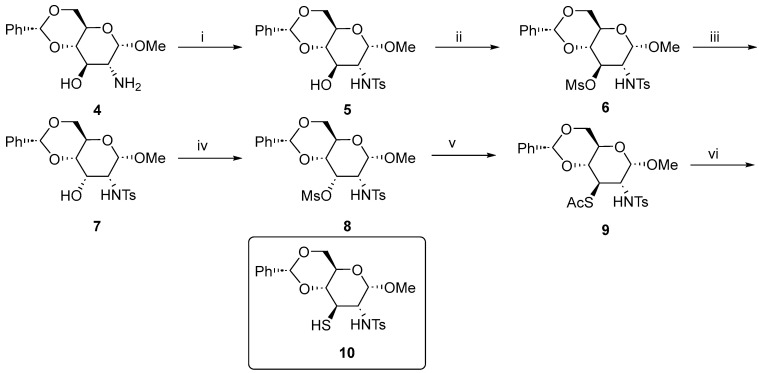
The synthesis of methyl 4,6-*O*-benzylidene-2,3-dideoxy-3-thio-2-*p*-toluenesulfonamido-α-d-glucopyranoside **10**. Reaction conditions: (i) *p-*TsCl, Na_2_CO_3_, 1,4-dioxane:H_2_O (1:1), 4 h, 92%; (ii) MsCl, Et_3_N, DCM. 0 °C to r.t., 3 h, 74%; (iii) NaOAc, 2-methoxyethanol:H_2_O (10:1), 110 °C, 46 h, 95%; (iv) MsCl, Et_3_N, DCM. 0 °C to r.t., 3 h, 90%; (v) KSAc, MeCN, 75 °C, 1.5 h, 89%; and (vi) LiAlH_4_, THF, 0 °C, 4 h, 41%.

At first, we choose the known [4] amino alcohol **4** as a most versatile starting material for the synthesis of all the ligands. The synthesis of methyl 4,6-*O*-benzylidene-2,3-dideoxy-3-thio-2-*p*-toluenesulfonamido-α-d-glucopyranoside **10** proceeded easily. The reaction at step (v) required some optimization, as the reaction in DMF gave a major product **9** contaminated with some side products; however, the same reaction performed in acetonitrile furnished **9** as a single compound (Scheme 1).

**Scheme 2:**
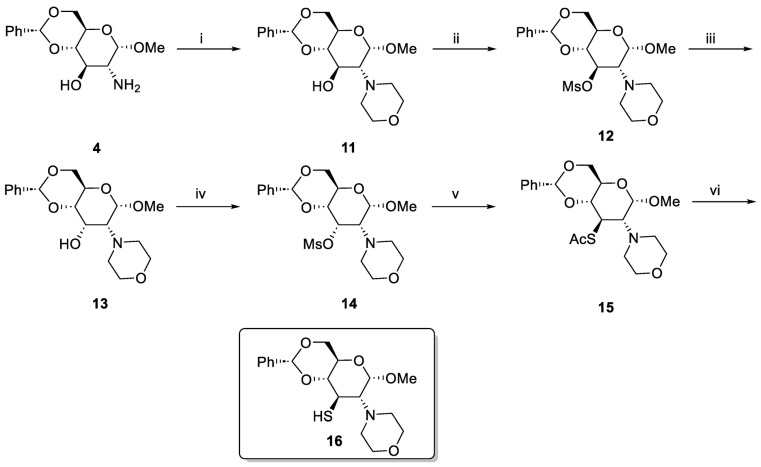
Synthesis of methyl 4,6-*O*-benzylidene-2,3-dideoxy-2-(4-morpholinyl)-3-thio-α-d-glucopyranoside **16**. Reaction conditions: (i) bis(2-bromoetyl) ether, MeCN, K_2_CO_3_, 80 °C, 20 h, 47%; (ii) MsCl, pyridine, r.t., 4 h, 73%; (iii) NaOAc, 2-methoxyethanol, 115 °C, 72 h, 46%; (iv) MsCl, pyridine, 4 h, r.t. 68%; (v) KSAc, DMF, 24 h, 100 °C, 70%; and (vi) LiAlH_4_, THF, 0 °C to r.t., 76%.

Successful synthesis of the sulfonamide **10** prompted us to apply the same method to the morpholine ligand **16**, belonging to the second series of ligands. The synthesis proceeded reasonably well; however, we encountered some problems. In particular, the inversion of C3 hydroxyl (step iii) was very slow and the product **13** was obtained with a moderate yield (Scheme 2).

Next, we applied this approach for the synthesis of a piperidine derivative, namely methyl 4,6-*O*-benzylidene-2,3-dideoxy-2-(1-piperidynyl)-3-thio-α-d-glucopyranoside. Unfortunately, even after the long optimization of the conditions, we could not achieve the inversion of C3 hydroxyl from the equatorial to axial position with a yield higher than 20% and the prolonged reaction times did not increase the conversion over 50% with a simultaneous decomposition of the product. Therefore, we abandoned this path for the synthesis.

We reached the conclusion that the presence of cyclic amine at C2 could be responsible for the problems with the inversion of the configuration at C3. Therefore, we decided to synthesize an analog of the amino alcohol **4**, the methyl 2-amino-4,6-*O*-benzylidene-2-deoxy-α-d-allopyranoside **20**.

We decided to try a not very popular but often highly efficient method: Lattrell–Dax epimerization [13], which has been successfully used in carbohydrate chemistry [14].

**Scheme 3:**
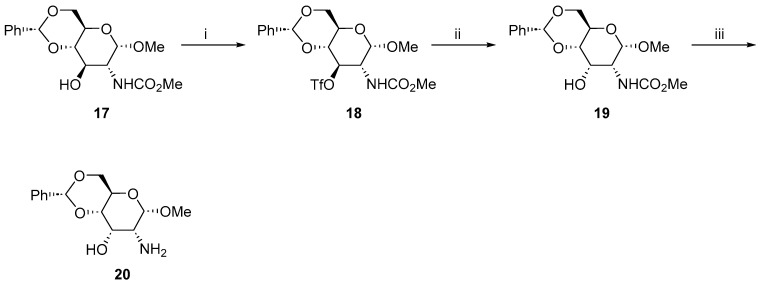
Synthesis of methyl 2-amino-4,6-*O*-benzylidene-2-deoxy-α-d-allopyranoside **20**. Reaction conditions: (i) Tf_2_O, pyridine, DCM, −20 °C, 2 h, 93%; (ii) NaNO_2_, DMF, r.t., 6 days, 67%; and (iii) 4N KOH, EtOH:2-methoxyethanol (2:1), 80 °C, 20 h, 52%.

We started from the carbamate **17**, an easily available precursor of amino alcohol **4** [4]. The synthesis of triflate **18** was straightforward and furnished the expected product with a high yield. The subsequent Lattrell–Dax epimerization proceeded smoothly, providing **19**, which was converted to the expected allopyranoside **20** (Scheme 3).

**Scheme 4:**
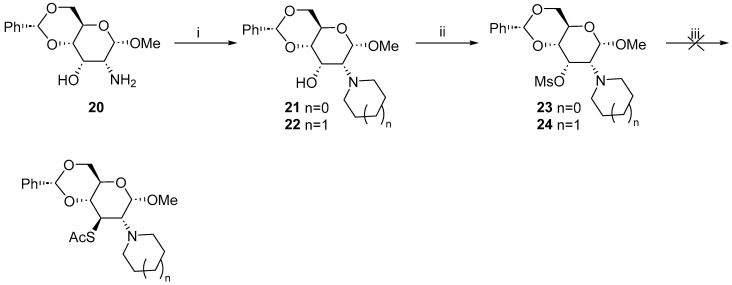
Attempted synthesis of pyrrolidinyl and piperidinyl-3-thio derivatives. Reaction conditions: (i) **21**: C_4_H_8_Br_2_, K_2_CO_3_, MeCN, reflux, 24 h, 88%; **22** C_5_H_10_Br_2_, K_2_CO_3_, MeCN, reflux, 40 h, 70%; (ii) MsCl, DCM, r.t., 4 h, **23** 52%, **24** 60%; and (iii) KSAc, DMF or MeCN or DMPU, reflux, 0%.

The formation of the pyrrolidine **21** and piperidine **22** derivatives was achieved using the respective dibromides; however, the mesylation of these compounds at C3 was only possible using pyridine and not triethylamine as a base. We then tried to convert both mesylates **23** and **24** into thioacetate. Much to our disappointment, treating the mesylate derivatives with potassium thioacetate failed to produce any product (Scheme 4).

The problems we encountered prompted us to cease our efforts toward the synthesis of pyrrolidinyl and piperidinyl derivatives and to expand the pool of C2 sulfonamides. 

**Scheme 5:**
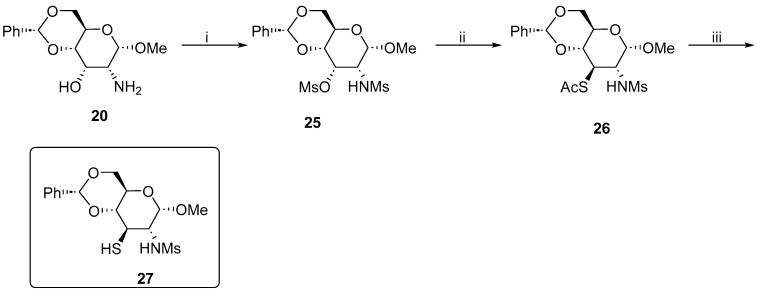
Synthesis of methyl 4,6-*O*-benzylidene-2,3-dideoxy-2-methanesulfonamido-3-thio-α-d-glucopyranoside **27**. Reaction conditions: (i) MsCl, pyridine, 0 °C, 24 h 75%; (ii) KSAc, DMPU, 100 °C, 6 h, 60%; and (iii) LiAlH_4_, 0 °C to r.t., 4 h, 65%.

First, the amino alcohol **20** was treated with an excess of methanesulfonyl chloride in the presence of pyridine. The resulting dimethanesulfonyl derivative **25** was subjected to the nucleophilic substitution reaction with potassium thioacetate in DMPU, which appeared to be the solvent of choice for this transformation. However, the expected product was accompanied by a by-product, presumably resulting from the elimination of the mesylate, and the mixture was inseparable at this stage. Fortunately, the reduction of thioacetate **26** furnished thiosulfonamide **27** in a good yield and the by-product could be removed at this final stage (Scheme 5). 

**Scheme 6:**
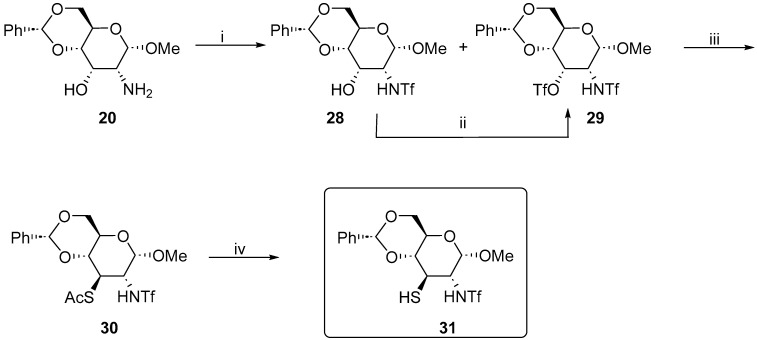
Synthesis of methyl 2,3-dideoxy-4,6-*O*-benzylidene-3-thio-2-trifloromethanesulfonamido-α-d-glucopyranoside **31**. Reaction conditions: (i) Tf_2_O, pyridine, DCM, −20 °C to 0 °C, 2 h, 51% (**29**), 34% (**28**); (ii) Tf_2_O, pyridine, DCM −20 °C, 2 h, 61%; (iii) KSAc, MeCN, 5 to 20 °C, 40%; and (iv) NaOMe, MeOH, r.t., 18 h, 76%.

The same strategy could be applied for the synthesis of trifluoromethylsulfonamide derivative **31**. The double protection of amino alcohol **20** could be achieved using an excess of triflic anhydride (3 equiv.) in DCM and pyridine (10 equiv.), resulting in a mixture of mono- and di-triflate products, approximately 40% to 60%. Fortunately, it was possible to separate these two compounds and the triflation of the remaining hydroxyl derivatives worked without any problems under similar conditions (Scheme 6).

The inversion procedure (KSAc, DMPU) developed for mesylate **25** did not work for the triflate **28**. The expected product **30** was obtained with a very low yield (15%), probably due to competitive elimination. The optimization of this reaction showed that acetonitrile is the solvent of choice for this transformation and **30** was obtained with 40% yield; however, the elimination was still a major problem. In the final conversion to free thiol **31** using standard approach, the reduction of thioacetate with LiAlH_4_ was unsuccessful and it could not be obtained by this method. We decided to hydrolyze the acetate under the Zemplen conditions [15]. The expected ligand **31** was obtained in a 76% yield.

Having to hand all the available ligands, we began studies on the enantioselective additions of diethylzinc to benzaldehyde. We started with the *p*-toluenesulfonamide ligand **10** using the titanium tetraisopropoxide-based method. Not very surprisingly, the enantioselectivity in the toluene was very poor (e.r. 57:43, *S*/*R*), and it was even lower when using methylene chloride as a solvent (e.r. 54.5:45.5). Supposedly, the required titanium–sulfur complex was not formed as expected; however, the ligands exhibited very good catalytic properties—the chemical yields of 1-phenylpropanol were high at –95% and 85%, respectively (Table 1, entries 1 and 2). Then, we tried the morpholine ligand **16** in the absence of Ti(O*i*Pr)_4_ and we obtained the expected product with a very good chemical yield of 83%, but again with a surprisingly low enantioselectivity, where the enantiomeric ratio was only 55:45, *R*/*S*, much lower than that for the respective amino alcohol reported by Davis and co-workers (Table 1, entry 3) [12]. It became obvious that the 3-thio-glucosamine ligands did not follow the expected modes of action; therefore, we decided to check the inductive properties of the sulfonamide ligands in the absence of Ti(O*i*Pr)_4_ Lewis acid. First, we tried the ligand **10** and noticed a substantial improvement of the enantiodiscrimination to 74.5:25.5, again with a good yield and the *S* enantiomer prevailing (Table 1, entry 4). The bulky trifluoromethanesulfonamide ligand **31** was less efficient and the observed e.r. was only 64:36 (Table 1, entry 5). Unexpectedly, the highest enantiomer ratio 80.5:19.5 was obtained for the methanesulfonamide derivative **27** (Table 1, entry 6).

The literature reports indicate that thioacetate derivative may also be an effective chiral ligand [16,17].

Therefore, we decided to check four intermediate compounds, **9**, **15**, **26**, and **30**, as ligands. They appeared to be much less efficient ligands than the respective thiols; the results are presented in Table 1, entries 7–10.

## 3. Discussion

The data presented in the introduction, indicating that amino thiols can be equally good, and often better, ligands in the addition of organozinc reagents to carbonyl compounds, are not confirmed in the case presented here of 3-thio-glucosamine derivatives. 

**Figure 2:**
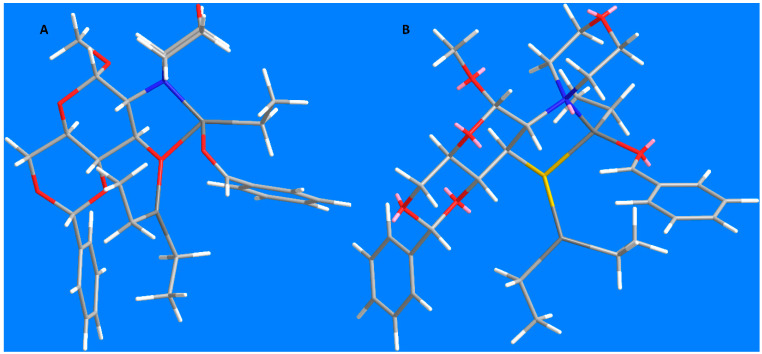
Transition state models: (**A**)—Davis’ ligand (ref. [12]); and (**B**)—ligand **16** (this work).

The low asymmetric induction of the reaction catalyzed by the sulfonamides in the presence of Ti(O*i*Pr)_4_ was not very surprising in the context of hard and soft acids and bases theory (Table 1, entries 1 and 2). However, the very low e.r. in the reaction catalyzed by the ligand **16** was unexpected (Table 1, entry 3). The earlier-reported hydroxy analogue [12] was quite efficient and we expected at least comparable enantioselectivity. Supposedly, the geometry of the transition state can be adversely influenced by the length of the zinc–sulfur bond, 2.2–2.4 Å vs. 1.9–2.2 Å for the zinc–oxygen bond. We performed MM2 energy minimalization in order to analyze the geometry of the most probable transition state based on the Noyori’s model (Figure 2).

The left image shows the model for the addition of Davis’ ligand [12], which exhibited good enantioselectivity (e.r. 82.5:17.5). As indicated in Noyori’s model, the ethyl group attached to the same zinc atom as the benzaldehyde efficiently hinders the rotation of the aldehyde, securing high enantiodiscrimination. A quite different situation is observed in the case of the thiol ligand **16**. That ethyl group is directed backwards relative to the aldehyde, which creates much less steric hindrance and does not prevent rotation of the aldehyde molecule. As a result, an attack on both faces of the carbonyl group is almost equally probable, what results in poor selectivity.

The reaction proceeded quite well for the sulfonamide ligands in the absence of the Lewis acid. The best results were obtained for the least sterically demanding mesyl derivative **27** and not for the highly hindered trifluorosulfonamide **31** (Table 1, entry 6 and 5), which, on the basis of our previous studies and the very high selectivity observed in the presence of the 3-hydroxy analog of **31**, should be the most efficient ligand. 

**Figure 3:**
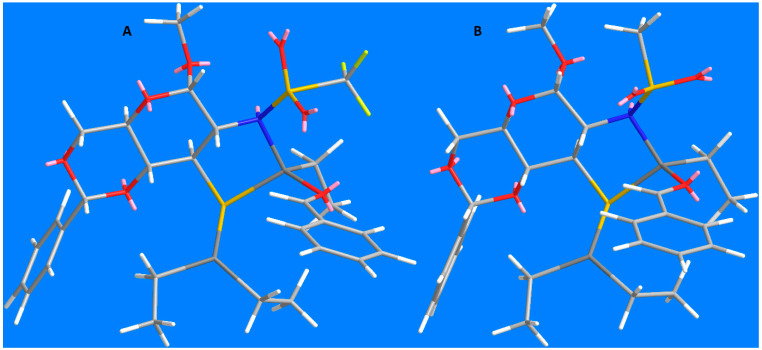
Transition state models: (**A**)—**31**; and (**B**)—**27**.

In the case of sulfonamides, the main factor influencing enantiodiscrimination is probably the interaction of the anomeric methoxyl group with the methyl- and trifluoromethylsulfonamide groups in **27** and **31**, respectively (Figure 3A,B). The bigger CF_3_ substituent has to be placed farther from the anomeric OCH_3_ (Figure 3A) and that allows for the easier rotation of the aldehyde, leading to diminished enantioselectivity. The smaller methanesulfonyl group locates itself in close proximity of the anomeric methoxy (Figure 3B) and hinders the rotation of the carbonyl group, securing higher enantiodiscrimination. 

Also, the results for the acetate derivatives tested suggest that various coordination modes are possible for the ligand–diethylzinc system, thus highly altering the transition state geometry and, as a consequence, the observed asymmetric induction. 

In conclusion, we have synthesized a series of novel 3-thio-derivatives of d-glucosamine. New compounds were applied as ligands for the diethylzinc addition to benzaldehyde and the products of the addition were obtained with a low to good enantiomeric ratio.

## 4. Material and Methods

Benzaldehyde was distilled under reduced pressure and stored under argon. Diethylzinc solution (1.1 M in toluene) was purchased from Sigma-Aldrich (St. Louis, MO, USA). All the reactions were performed under an argon atmosphere in oven-dried glassware using the Schlenk technique when necessary. The ^1^H and ^13^C NMR spectra were recorded in CDCl_3_ using a Bruker AVANCE 300 MHz spectrometer (Bruker, Billerica, MA, USA). All the chemical shifts are quoted in parts per million relatives to tetramethylsilane (δ, 0.00 ppm) as the internal standard. The ^1^H-NMR splitting patterns are abbreviated as follows: singlet (s), doublet (d), triplet (t), quartet (q), dd (doublet of doublets), and m (multiplets). The high-resolution mass spectra were recorded on a Quattro LC Micromass, LCT Micromass TOF HiRes, and Shimadzu LCMS-9030 apparatus. Flash column chromatography was performed on silica gel (Kieselgel-60, Merck, 230–400 mesh). TLC analyses were performed on 250 μm Silica Gel 60 F254 plates (Merck, Rahway, NJ, USA) and visualized by the quenching of the UV fluorescence at 254 nm or by staining with molybdic acid–cerium (II) sulphate solution. High-performance liquid chromatography was conducted on a Knauer chromatograph (Berlin, Germany) equipped with a diode array detector using a Diacel Chiralcel OD-H column (Osaka, Japan). Compounds **4**, **5** and **17** were synthesized according to published procedures [4].

The Supplementary Material (Figures S1–S27) contains the ^1^H and ^13^C NMR spectra as PDF copies.

### 4.1. General Procedure for Diethylzinc Addition to Benzaldehyde

In the Schlenk tube equipped with a stirring element, a ligand was placed (0.1 equiv.), and the tube was flushed three times with argon. Then, the solvent was added (1.25 mL). After 10 min, 1.1 M ZnEt_2_ in toluene (2 equiv., 0.945 mL) was added. After 30 min of stirring at room temperature, freshly distilled benzaldehyde (0.5 mmol, 51 μL) was added, and then the reaction was stirred at the indicated time until TLC showed the disappearance of the aldehyde. The reaction was diluted with diethyl ether and quenched with 1 M HCl. The organic layer was separated, and the aqueous layer was extracted twice with ether. The combined organic layers were then concentrated and purified by column chromatography (hexane-EtOAc 7:3) to give 1-phenyl-1-propanol as a colorless oil. The ^1^H-NMR (300 MHz, CDCl_3_) δ 7.43–7.23 (m, 5H, C_6_H_5_), 4.62 (t, J 6.6 Hz, 1H, Ph*CH*(OH)Et), 1.90–1.70 (m, 2H, CH_2_), and 0.95 (t, J 7.4 Hz, 3H, CH_3_).

### 4.2. General Procedure for Diethylzinc Addition to Benzaldehyde in the Presence of Ti(OiPr_4_)

In the Schlenk tube equipped with a stirring element, a ligand was placed (0.1 equiv.), and the tube was flushed three times with argon. Then, methylene chloride was added (3 mL), followed by Ti(O*i*Pr)_4_ (1.4 equiv., 0.24 mL). After 1.5 h, the reaction mixture was cooled to 0 °C and 1.1 M ZnEt_2_ (3 equiv., 1.35 mL) was added. After 45 min, an aldehyde (0.5 mmol) was added and stirred at 0 °C for an additional 30 min, then allowed to warm up to room temperature. The reaction was stirred at this temperature until TLC showed the disappearance of the aldehyde. The reaction was diluted with diethyl ether and quenched with 1 M HCl. The organic layer was separated, and the aqueous layer was extracted twice with ether. The combined organic layers were then concentrated and purified by column chromatography (hexane-EtOAc 7:3) to give 1-phenyl-1-propanol as a colorless oil. The ^1^H-NMR (300 MHz, CDCl_3_) δ 7.43–7.23 (m, 5H, C_6_H_5_), 4.62 (t, J 6.6 Hz, 1H, Ph*CH*(OH)Et), 1.90–1.70 (m, 2H, CH_2_), and 0.95 (t, J 7.4 Hz, 3H, CH_3_). 

### 4.3. Methyl 4,6-O-Benzylidene-2-deoxy-3-O-methanesulfonyl-2-p-toluenesulfonamide-α-d-glucopyranoside (***6***)

To the stirred solution of **5** (3.4 g, 7.9 mmol) in DCM (60 mL) at 0 °C, triethylamine (1.6 g, 15.8 mmol) and MsCl (1.22 mL, 15.8 mmol) were added. The reaction was left at 0 °C for 30 min, then stirred at room temperature for 3 h. The volatiles were removed under a vacuum. The organic phases were washed with water and brine and dried over anhydrous MgSO_4_. The crude was purified by column chromatography (hexane-EtOAc 3:2) to yield methanesulfonamide **6** as a white solid (2.9 g, 74%). The ^1^H-NMR (300 MHz, CDCl_3_) δ 7.84 (d, J 8.2 Hz, 2H), 7.51–7.30 (m, 7H), 5.52 (s, 1H), 5.21 (d, J 9.8 Hz, 1H), 4.82–4.63 (m, 2H), 4.31 (dd, J 9.8, 4.2 Hz, 1H), 3.91–3.79 (m, 1H), 3.80–3.63 (m, 2H), 3.62–3.50 (m, 1H), 3.39 (s, 3H), 2.93 (s, 3H), and 2.45 (s, 3H). The ^13^C-NMR (75 MHz, CDCl_3_) δ 144.0, 137.1, 136.5, 129.9, 129.3, 128.4, 127.4, 126.0, 101.8, 99.7, 79.1, 68.7, 62.7, 56.3, 55.9, 52.6, 38.9, and 21.6. HRMS (ESI-TOF) calcd. for C_22_H_27_NO_9_S_2_Na [M + Na]^+^: 536.1023; found 536.1013.

### 4.4. Methyl 4,6-O-Benzylidene-2-deoxy-2-p-toluenesulfonamide-α-d-allopyranoside (***7***)

To the stirred solution of a substrate, **6** (2.6 g, 5 mmol) in 2-methoxyethanol/H_2_O (10:1, 83 mL), sodium acetate anhydrous (4 g, 48 mmol) was added. The reaction was stirred at 110 °C for 46 h. Then, the reaction was cooled to room temperature, the solvent was removed under a vacuum, and the crude was diluted with DCM. The organic phase was washed with water and brine, and dried over anhydrous MgSO_4_. The yellow residue was purified by column chromatography (hexane-EtOAc 3:2) to give **7** as a white solid (2.1 g, 95%). The ^1^H-NMR (300 MHz, CDCl_3_) δ 7.81–7.61 (m, 2H), 7.40–7.16 (m, 5H), 7.05–6.93 (m, 2H), 5.71 (d, J 9.4 Hz, 1H), 5.48 (s, 1H), 4.68 (d, J 1.2, 0.6 Hz, 1H), 4.27 (dd, J 10.1, 4.7 Hz, 1H), 4.08 (ddd, J 5.7, 2.9, 1.2 Hz, 1H), 4.02 (dd, J 9.8, 4.1 Hz, 1H), 3.94–3.83 (m, 2H), 3.76 (t, J 9.9 Hz, 1H), 3.47 (s, 3H), 2.83 (d, J 5.8 Hz, 1H), and 2.29 (s, 3H). The ^13^C-NMR (75 MHz, CDCl_3_) δ 143.1, 137.5, 137.2, 129.3, 128.9, 128.1, 127.4, 126.2, 101.9, 101.7, 73.7, 70.3, 69.1, 59.6, 55.8, 53.6, and 21.6. HRMS (ESI-TOF) calcd. for C_21_H_25_NO_7_SNa [M + Na]^+^: 458.1249; found 458.1249.

### 4.5. Methyl 4,6-O-Benzylidene-2-deoxy-3-O-methanesulfonyl-2-p-toluenesulfonamido-α-d-allopyranoside (***8***)

To the stirred solution of **7** (2.1 g, 4.9 mmol) in DCM (60 mL) at 0 °C, triethylamine (1.38 mL, 9.9 mmol) and mesyl chloride (0.7 mL, 9.9 mmol) were added. The reaction was left at 0 °C for 30 min, then stirred at room temperature for 3 h. The solvent was removed under a vacuum, and the organic phase was washed with water and brine and dried over anhydrous MgSO_4_. The crude was purified by column chromatography (hexane-EtOAc 3:2) to yield methanesulfonamide **8** as a white solid (2.3 g, 90%). The ^1^H-NMR (300 MHz, CDCl_3_) δ 7.75 (d, J 8.3 Hz, 2H), 7.45–7.25 (m, 5H), 7.16–6.98 (m, 2H), 5.58 (d, J 8.8 Hz, 1H), 5.51 (s, 1H), 4.83 (t, J 1.2 Hz, 1H), 4.77 (dd, J 2.7, 1.3 Hz, 1H), 4.33 (dd, J 9.4, 3.2 Hz, 1H), 4.04–3.90 (m, 3H), 3.87–3.72 (m, 1H), 3.49 (s, 3H), 3.15 (s, J 4.9 Hz, 3H), and 2.36 (s, 3H). The ^13^C-NMR (75 MHz, CDCl_3_) δ 143.5, 136.8, 136.7, 129.4, 129.05, 128.0, 127.4, 126.1, 101.7, 99.3, 75.9, 72.8, 68.9, 58.9, 56.0, 51.6, 37.9, and 21.5. HRMS (ESI-TOF) calcd. for C_22_H_27_NO_9_S_2_Na [M + Na]^+^: 536.1025; found 536.1038.

### 4.6. Methyl 4,6-O-Benzylidene-2,3-dideoxy-3-thioacetyl-2-p-toluenesulfonamido-α-d-glucopyranoside (***9***)

Mesylate **8** (200 mg, 0.38 mmol) was stirred in acetonitrile (8 mL) under argon, and then potassium thioacetate (222 mg, 1.94 mmol) was added. The reaction was stirred at 75 °C for 1.5 h. Then, the solvent was removed under a vacuum, and the crude was diluted with dichloromethane. The organic phases were washed with water and brine, and dried over anhydrous MgSO_4_. The brown residue was purified by column chromatography (hexane-EtOAc 3:2) to give thioacetate **9** as a yellow solid (171 mg, 89%). The ^1^H-NMR (300 MHz, CDCl_3_) δ 7.81–7.70 (m, 2H), 7.42–7.23 (m, 5H), 7.18–7.00 (m, 2H), 5.92 (d, J 9.2 Hz, 1H), 5.52 (s, 1H), 4.59 (q, J 0.8 Hz, 1H), 4.26 (dd, J 10.0, 4.2 Hz, 1H), 4.06–3.89 (m, 3H), 3.–3.63 (m, 2H), 3.48 (s, 3H), 2.35 (s, 3H), and 2.32 (s, 3H). The ^13^C NMR (75 MHz, CDCl_3_) δ 191.7, 143.0, 138.0, 136.9, 129.4, 129.0, 128.1, 127.3, 126.2, 101.8, 101.6, 75.2, 69.0, 59.5, 56.1, 53.2, 46.0, 30.4, and 21.6. HRMS (ESI-TOF) calcd. for C_23_H_27_NO_7_S_2_[M + Na]^+^: 516.1127; found 516.1138.

### 4.7. Methyl 4,6-O-Benzylidene-2,3-dideoxy-3-thio-2-p-toluenesulfonamido-α-d-glucopyranoside (***10***)

Lithium aluminum hydride (202 mg, 5.32 mmol) was stirred in dry THF (12 mL) at 0 °C, and then thioacetate substrate **9** (376 mg, 0.76 mmol) in dry THF (12 mL) was added. The reaction was stirred at 0 °C for 30 min, then continued stirring at room temperature for 4 h. After completion of the reaction, the reaction was worked up according to the Fieser workup: diluted with diethyl ether, followed by water, 15% aq. NaOH solution, water again, then dried with anhydrous MgSO_4_. The organic solvent was evaporated under reduced pressure, and the crude was purified by column chromatography (hexane-EtOAc 7:3) to yield **10** as a white solid (145 mg, 41%). The ^1^H-NMR (300 MHz, CDCl_3_) δ 7.79–7.54 (m, 2H), 7.44–7.24 (m, 5H), 7.08–6.95 (m, 2H), 5.83 (d, J 9.5 Hz, 1H), 5.52 (s, 1H), 4.75 (q, J 0.9 Hz, 1H), 4.27 (dd, J 10.2, 4.9 Hz, 1H), 4.13 (dd, J 9.9, 4.2 Hz, 1H), and 4.04–3.87 (m, 2H), 3.79 (t, J 10.1 Hz), 3.45 (s, 3H), 3.36 (ddd, J 9.9, 2.4, 0.9 Hz, 1H), 2.34 (s, 3H), 2.13 (d, J 9.8 Hz, 1H). The ^13^C-NMR (75 MHz, CDCl_3_) δ 143.0, 137.4, 136.9, 129.2, 128.9, 128.0, 127.3, 126.2, 102.9, 101.8, 72.7, 69.0, 59.7, 55.8, 55.4, 41.8, and 21.5. HRMS (ESI-TOF) calcd. for C_21_H_25_NO_6_S_2_Na [M + Na]^+^: 474.1021; found 474.1017.

### 4.8. Methyl 4,6-O-Benzylidene-2-deoxy-2-(4-morpholinyl)-α-d-glucopyranoside (***11***)

To the stirred solution of amino alcohol **4** (1.83 g, 6.624 mmol) solution in acetonitrile, potassium carbonate (1.83 g, 13.24 mmol) and bis(2-bromoetyl)-diethyl ether (1.24 mL, 9.936 mmol) were added. The reaction stirred for 20 h under reflux. The solvent was evaporated under reduced pressure, and the crude was diluted in DCM. The organic phase was washed with water and brine, and dried over anhydrous MgSO_4_. Purification by flash column chromatography (hexane-EtOAc 1:5) gave **11** as a white solid (1.1 g, 47%). The ^1^H NMR (300 MHz, CDCl_3_) δ 7.59–7.46 (m, 2H), 7.44–7.32 (m, 3H), 5.59 (s, 1H), 4.87 (d, J 3.1 Hz, 1H), 4.32–4.26 (m, 1H), 4.20 (dd, J 10.5, 8.7 Hz, 1H), 3.90–3.77 (m, 2H), 3.70–3.63 (m, 4H), 3.59 (t, J 8.9 Hz, 1H), 3.42 (s, 3H), 3.13 (d, J 0.9 Hz, 1H), 2.86 (dt, J 6.0, 3.1 Hz, 4H), and 2.72 (dd, J 10.6, 3.2 Hz, 1H). 

### 4.9. Methyl 4,6-O-Benzylidene-2-deoxy-3-O-methanesulfonyl-2-(4-morpholinyl)-α-d-glucopyranoside (***12***)

The substrate **11** (1000 mg, 2.84 mmol) was stirred in dry dichloromethane (10 mL) and pyridine (10 mL) for 5 min at room temperature, and then mesyl chloride (977.68 mg, 8.53 mmol) was added dropwise. The reaction was stirred under argon for 4 h, then quenched with methanol. The solvent was removed under reduced pressure and diluted with dichloromethane. The organic phases were washed with water and brine and dried over anhydrous MgSO_4_. The crude was purified by column chromatography (hexane-EtOAc 1:2) to give mesylate **12** as a white solid (900 mg, 73%). The ^1^H NMR (300 MHz, CDCl_3_) δ 7.52–7.46 (m, 2H), 7.42–7.37 (m, 3H), 5.57 (s, 1H), 5.17 (dd, J 10.8, 9.0 Hz, 1H), 4.90 (d, J 3.2 Hz, 1H), 4.32 (dd, J 10.1, 4.6 Hz, 1H), 3.91 (td, J 9.8, 4.5 Hz, 1H), 3.83–3.73 (m, 2H), 3.73–3.64 (m, 4H), 3.43 (s, 3H), 2.98 (s, 3H), 2.96 (d, J 3.2 Hz, 1H), and 2.91 (m, 4H). The ^13^C-NMR (75 MHz, CDCl_3_) δ 136.7, 129.4, 128.4, 126.1, 101.9, 100.4, 81.0, 76.8, 69.1, 67.8, 67.1, 62.4, 54.8, 50.5, and 39.2. HRMS (ESI-TOF) calcd. for C_19_H_27_NO_8_SNa [M + Na]^+^: 452.1355; found 452.1364.

### 4.10. Methyl 4,6-O-Benzylidene-2-deoxy-2-(4-morpholinyl)-α-d-allopyranoside (***13***)

The substrate **12** (1.15 g, 2.67 mmol) was suspended in 2-methoxyethanol/H_2_O (10:1, 36 mL) and reacted with anhydrous sodium acetate (1.7 g, 20 mmol) at 115–125 °C for 72–120 h. The solvent was removed under reduced pressure, the residue was diluted with DCM, and the organic phases were washed with water and brine, and dried over anhydrous MgSO_4_. The crude was purified by column chromatography (4% MeOH-DCM) to give hydroxyl **13** as a white solid (440 mg, 46%). The ^1^H NMR (300 MHz, CDCl_3_) δ 7.50–7.44 (m, 2H) 7.42–7.36 (m, 3H), 5.51 (s, 1H), 4.55 (d, J 1.9 Hz, 1H), 4.40–4.28 (m, 2H), 4.26–4.11 (m, 2H), 3.79–3.64 (m, 6H), 3.43 (s, 3H), 3.09–2.94 (m, 3H), and 2.87–2.78 (m, 2H). The ^13^C-NMR (75 MHz, CDCl_3_) δ 137.7, 129.1, 128.4, 126.2, 102.6, 101.5, 78.7, 69.8, 68.8, 67.6, 64.9, 60.0, 55.3, and 52.9. HRMS (ESI-TOF) calcd. for C_18_H_26_NO_6_ [M + H]^+^: 352.1760; found 352.1749.

### 4.11. Methyl 4,6-O-Benzylidene-2-deoxy-3-O-methanesulfonyl-2-(4-morpholinyl)-α-d-allopyranoside (***14***) 

The substrate **13** (600 mg, 1.7 mmol) was stirred in dry DCM (6 mL) and pyridine (6 mL) for 5 min at room temperature, and then mesyl chloride (586 mg, 5.12 mmol) was added dropwise. The reaction was stirred under argon for 4 h, then quenched with MeOH. The solvent was removed under reduced pressure and diluted with DCM. The organic phases were washed with water and brine, and dried over anhydrous MgSO_4_. The crude was purified by column chromatography (hexane-EtOAc 1:2) to give mesylate **14** as a white solid (500 mg, 68%). The ^1^H-NMR (300 MHz, CDCl_3_) δ 7.51–7.43 (m, 2H), 7.43–7.35 (m, 3H), 5.52 (s, 1H), 5.05 (dd, J 2.5, 1.2 Hz, 1H), 4.72 (p, J 0.6 Hz, 1H), 4.42–4.31 (m, 2H), 4.16–4.06 (m, 1H), 3.76–3.67 (m, 5H) 3.41 (s, 3H), 3.18 (ddd, J 4.6, 2.5, 0.8 Hz, 1H) 3.11 (s, 3H), 3.11–3.04 (m, 2H), and 2.79 (m, 2H). The ^13^C-NMR (75 MHz, CDCl_3_) δ 137.4, 129.2, 128.4, 126.1, 102.7, 98.6, 77.6, 75.9, 69.5, 67.4, 63.3, 59.1, 55.5, 52.9, and 38.7. HRMS (ESI-TOF) calcd. for C_19_H_27_NO_8_SNa [M + Na]^+^: 452.1355; found 452.1369. 

### 4.12. Methyl 4,6-O-Benzylidene-2,3-dideoxy-2-(4-morpholinyl)-3-thioacetyl-α-d-glucopyranoside (***15***)

Mesylate **14** (311 mg, 0.72 mmol) was diluted in anhydrous DMF (20 mL) under argon, and then potassium thioacetate (331 mg, 2.89 mmol) was added. The reaction was stirred at 100 °C for 24 h. Then, the solvent was removed under a vacuum, and the crude was diluted with EtOAc. The organic phases were washed with water, saturated aq. NH_4_Cl solution, and brine, and dried over anhydrous MgSO_4_. The brown residue was purified by column chromatography (hexane-EtOAc 7:3) to give thioacetate **15** as a white solid (210 mg, 70%). The ^1^H-NMR (300 MHz, CDCl_3_) δ 7.43–7.34 (m, 5H), 5.46 (s, 1H), 4.54 (dt, J 0.9, 0.5 Hz, 1H), 4.43 (td, J 9.9, 5.5 Hz, 1H), 4.31 (d, J 1.2 Hz, 1H), 4.28 (t, J 5.0 Hz, 1H), 3.93 (dd, J 9.6, 3.8 Hz, 1H), 3.81–3.70 (m, 4H), 3.72–3.60 (m, 1H), 3.39 (s, 3H), 3.10–2.99 (m, 2H), 2.98 (dd, J 1.7 Hz, 1H), 2.88 (m, 2H), and 2.40 (s, 3H). The ^13^C-NMR (75 MHz, CDCl_3_) δ 193.2, 137.6, 129.2, 128.4, 126.2, 102.6, 100.6, 79.9, 69.5, 67.5, 64.7, 58.8, 55.4, 53.5, 43.7, and 30.4. HRMS (ESI-TOF) calcd. for C_20_H_27_NO_6_SNa [M + Na]^+^: 432.1457; found 432.1472.

### 4.13. Methyl 4,6-O-Benzylidene-2,3-dideoxy-2-(4-morpholinyl)-3-thio-α-d-glucopyranoside (***16***)

Lithium aluminum hydride (130 mg, 3.42 mmol) was stirred in dry THF (7 mL) at 0 °C, and then thioacetate **16** (175 mg, 0.42 mmol) in dry THF (7 mL) was added. The reaction was stirred at 0 °C for 30 min, then continued at room temperature for 2.5 h. After completion of the reaction, the reaction was quenched according to the Fieser workup: diluted with diethyl ether, followed by water, 15% aq. NaOH solution (0.5 mL), water again, then dried over anhydrous MgSO_4_. The organic solvent was evaporated under reduced pressure, and the crude was purified by column chromatography (hexane-Et_2_O 1:1) to obtain ligand **16** as a white solid (120 mg, 76%). The ^1^H NMR (300 MHz, CDCl_3_) δ 7.53–7.43 (m, 2H), 7.42–7.35 (m, 3H), 5.51 (s, 1H), 4.69 (d, J 1.4 Hz, 1H), 4.43–4.23 (m, 3H), 3.81–3.61 (m, 5H), 3.58 (ddd, J 8.5, 2.7, 1.5 Hz, 1H), 3.39 (s, 3H), 3.15 (dd, J 4.3, 2.7 Hz, 1H), 3.10–2.95 (m, 2H), 2.95–2.72 (m, 2H), and 2.24 (d, J 8.5 Hz, 1H). The ^13^C NMR (75 MHz, CDCl_3_) δ 137.7, 129.1, 128.4, 126.2, 102.7, 102.3, 77.8, 69.7, 67.7, 66.6, 59.8, 55.3, 52.8, and 39.6. HRMS (ESI-TOF) calcd. for C_18_H_25_NO_5_SNa [M + Na]^+^: 390.1351; found 390.1367.

### 4.14. Methyl 4,6-O-Benzylidene-2-deoxy-2-methoxycarbonylamido-3-O-trifluoromethanesulfonyl-α-d-glucopyranoside (***18***)

The substrate **17** (4 g, 11.7 mmol) was dissolved in dry dichloromethane (117 mL) and pyridine (9.4 mL, 117 mmol) was added at −20 °C. The reaction was stirred for 10 min before triflic anhydride (7.4 g, 4.36 mL, 35.3 mmol) was added dropwise under argon and left at that temperature for 2 h. The reaction was then quenched with saturated aq. NaHCO_3,_ and the organic phase were separated. The organic phase was washed three times with 3% aq. CuSO_4_ solution, then with water and brine, and dried over anhydrous MgSO_4_. The crude was purified by column chromatography (hexane-EtOAc 1:2) to provide the triflate **18** a white–yellowish solid (5.1 g, 94% yield). The ^1^H-NMR (300 MHz, CDCl_3_) δ 7.50–7.43 (m, 2H), 7.39–7.33 (m, 3H), 5.59 (s, 1H), 5.15 (d, J 10.4 Hz, 1H), 5.03–4.90 (m, 1H), 4.78 (d, J 3.7 Hz, 1H), 4.37–4.20 (m, 2H), 3.88–3.77 (m, 3H), 3.72 (s, 3H), and 3.41 (s, 3H). The ^13^C-NMR (75 MHz, CDCl_3_) δ 156.4, 136.4, 129.2, 128.3, 125.9, 101.4, 99.7, 84.4, 78.5, 68.6, 63.0, 55.7, 53.8, and 52.8. HRMS (ESI-TOF) calcd. for C_19_H_27_NO_5_Na [M + Na]^+^: 372.17814; found 372.17850.

### 4.15. Methyl 4,6-O-Benzylidene-2-deoxy-2-methoxycarbonylamido-α-d-allopyranoside (***19***)

The triflate **18** (5.2 g, 11.03 mmol) was dissolved in dry DMF (55 mL), and sodium nitrite (5.2 g, 77.2 mmol) was added. The reaction was stirred at room temperature under argon for six days before the solvent was evaporated under a vacuum and the residue was extracted with ethyl acetate, and the organic phase was washed with water and brine, and dried over anhydrous MgSO_4_. The crude was purified by column chromatography (hexane-EtOAc 1:2) to provide the hydroxyl **19** as a white solid (3 g, 67%). The ^1^H NMR (300 MHz, CDCl_3_) δ 7.54–7.44 (m, 2H), 7.44–7.31 (m, 3H), 5.61 (s, 1H), 5.52 (d, J 9.6 Hz, 1H), 4.76 (d, J 4.0 Hz, 1H), 4.38 (dd J 10.4 Hz, 1H) 4.25–4.18 (m, 1H), 4.18–4.07 (m, 1H), 3.99 (dd, J 9.4, 3.9 Hz, 1H), 3.79 (t, J 10.3 Hz, 1H), 3.71 (s, 3H), 3.64 (dd, J 9.4, 2.8 Hz, 1H), 3.45 (s, 3H), and 2.62 (d, J 6.5 Hz, 1H). The ^13^C NMR (75 MHz, CDCl_3_) δ 156.5, 137.1, 129.3, 128.4, 126.3, 102.0, 99.5, 78.6, 69.2, 68.4, 57.5, 56.3, 52.4, and 51.3. HRMS (ESI-TOF) calcd. for C_16_H_21_NO_7_Na [M + Na]^+^: 362.1202; found 362.12198.

### 4.16. Methyl 4,6-O-Benzylidene-2-deoxy-2-amino-α-d-allopyranoside (***20***)

The substrate **19** (800 mg, 2.37 mmol) was dissolved in a solution of 4 N KOH in ethanol:2-methoxyethanol (2:1, 0.15 M, 15.7 mL) and the mixture was refluxed overnight. The dark brown residue was neutralized with a few amounts of 1 N HCl and extracted with chloroform three times. The organic phase was separated and washed with water and brine, and dried over anhydrous MgSO_4_. The crude was purified by column chromatography (EtOAc-MeOH 5:2) to afford the amino alcohol **20** as a white solid (332 mg, 50%). The ^1^H NMR (300 MHz, CDCl_3_) δ 7.54–7.47 (m, 2H), 7.40–7.30 (m, 3H), 5.57 (s, 1H), 4.64 (dd, J 3.8, 0.7 Hz, 1H), 4.36 (dd, J 10.2, 5.1 Hz, 1H), 4.15–3.99 (m, 2H), 3.75 (t, J 10.3 Hz, 1H), 3.52 (dd, J 9.7, 2.7 Hz, 1H), 3.44 (s, 3H), 2.94 (t, J 3.8, 3.1 Hz, 1H), and 2.06 (s, 3H). The ^13^C NMR (75 MHz, CDCl_3_) δ 137.3, 129.2, 128.3, 126.3, 102.1, 102.0, 79.4, 70.7, 69.4, 57.4, 56.2, and 52.5. HRMS (ESI-TOF) calcd. for C_14_H_20_NO_5_ [M + H]^+^: 282.13360; found 282.13348.

### 4.17. Methyl 4,6-O-Benzylidene-2-deoxy-2-(1-pyrrolidinyl)-α-d-allopyranoside (***21***)

To the stirred solution of amino alcohol substrate **20** (150 mg, 0.54 mmol) in acetonitrile (5 mL), potassium carbonate (149 mg, 1.08 mmol), and 1,4-dibromobutane (350 mg, 1.62 mmol) were added. The reaction was stirred for 24 h under reflux. The solvent was evaporated under reduced pressure, and the crude was diluted in chloroform. The organic phase was washed with water and brine, and dried over anhydrous MgSO_4_. Purification by flash column chromatography (EtOAc-MeOH 5:2) gave **21** as a white solid (160 mg, 88%). The ^1^H NMR (300 MHz, CDCl_3_) δ 7.57–7.47 (m, 2H), 7.44–7.26 (m, 3H), 5.59 (s, 1H), 4.79 (d, J 3.5, 0.8 Hz, 1H), 4.37 (dd, J 10.3, 5.3 Hz, 1H), 4.31 (t, J 2.5 Hz, 1H), 4.21 (td, J 9.8, 5.1 Hz, 1H), 3.76 (t, J 10.2 Hz, 1H), 3.54 (dd, J 9.7, 2.7 Hz, 1H), 3.45 (s, 3H), 2.82–2.68 (m, 2H), 2.66–2.52 (m, 2H), 2.37–2.28 (t, J 3.5, 2.8 Hz, 1H), and 1.95–1.82 (m, 4H). The ^13^C NMR (75 MHz, CDCl_3_) δ 137.3, 129.0, 128.2, 126.4, 102.1, 99.9, 79.6, 69.3, 67.1, 66.6, 57.8, 55.7, 51.5, and 23.0. HRMS (ESI-TOF) calcd. for C_18_H_25_NO_5_SNa [M + Na]^+^: 358.16249; found 358.16291.

### 4.18. Methyl 4,6-O-Benzylidene-2-deoxy-2-(1-piperidinyl)-α-d-allopyranoside (***22***)

To the stirred solution of **20** (400 mg, 1.44 mmol) in acetonitrile (18 mL), potassium carbonate (397 mg, 4.32 mmol) and 1,5-dibromobutane (0.58 mL, 993 mg, 4.32 mmol) were added. The reaction was stirred for 40 h under reflux. The solvent was evaporated under reduced pressure, and the crude was diluted in chloroform. The organic phase was washed with water and brine, and dried over anhydrous MgSO_4_. Purification by flash column chromatography (EtOAc-MeOH 5:2) gave **22** as a white solid (355 mg, 70%). The ^1^H NMR (300 MHz, CDCl_3_) δ 7.58–7.46 (m, 2H), 7.40–7.30 (m, 3H), 5.58 (s, 1H), 4.90 (d, J 3.4, 1H), 4.43 (t, J 2.7, 2.6 Hz, 1H), 4.36 (dd, J 10.1, 5.2 Hz, 1H), 4.20 (td, J 10.0, 5.1 Hz, 1H), 3.75 (t, J 10.2 Hz, 1H), 3.49 (dd, J 9.7, 2.7 Hz, 1H), 3.43 (s, 3H), 2.76–2.61 (m, 2H), 2.55–2.40 (m, 2H), 2.39 (t, J 3.5, 2.7 Hz, 1H), 1.70–1.60 (m, 4H), and 1.48 (m, J 6.1 Hz, 2H). The ^13^C NMR (75 MHz, CDCl_3_) δ 135.4, 129.1, 128.3, 126.4, 102.2, 99.1, 80.0, 69.4, 66.1, 65.1, 57.8, 55.5, 51.1, 28.5, and 25.8. HRMS (ESI-TOF) calcd. for C_19_H_27_NO_5_Na [M + Na]^+^: 372.17814; found 372.1770. 

### 4.19. Methyl 4,6-O-Benzylidene-2-deoxy-3-O-methanesulfonyl-2-(1-pyrrolidinyl)-α-d-allopyranoside (***23***)

The pyrrolidine substrate **21** (80 mg, 0.23 mmol) was stirred in 1:1 dry DCM:pyridine (1.5 mL) for 5 min at 0 °C, and then mesyl chloride (81 mg, 0.05 mL, 0.71 mmol) was added dropwise. The reaction was stirred under argon for 4 h at room temperature, then quenched with methanol. The solvent was removed under reduced pressure and diluted with dichloromethane. The organic phases were washed with water and brine, and dried over anhydrous MgSO_4_. The crude was purified by column chromatography (hexane-EtOAc 1:4) to give product **23** as a white solid (50 mg, 52%). The ^1^H NMR (300 MHz, CDCl_3_) δ 7.53–7.45 (m, 2H), 7.36 (dd, J 5.1, 1.8 Hz, 3H), 5.60 (s, 1H), 5.37 (t, J 2.8 Hz, 1H), 4.78 (d, J 3.7 Hz, 1H), 4.37 (dd, J 10.3, 5.2 Hz, 1H), 4.21 (td, J 10.0, 5.2 Hz, 1H), 3.79–3.68 (m, 2H), 3.44 (s, 3H), 3.05 (s, 3H), 2.79 (m, 2H), 2.58 (m, 2H), 2.46 (t, J 3.3 Hz, 1H), and 1.84 (ddd, J 6.7, 4.6, 2.1 Hz, 4H). The ^13^C NMR (75 MHz, CDCl_3_) δ 136.9, 129.2, 128.4, 126.1, 101.8, 99.2, 75.8, 69.2, 65.7, 58.1, 55.8, 51.9, 39.5, and 23.0. HRMS (ESI-TOF) calcd. for C_19_H_27_NO_7_SNa [M + Na]^+^: 436.14004; found 436.13993.

### 4.20. Methyl 4,6-O-Benzylidene-2-deoxy-3-O-methanesulfonyl-2-(1-piperidinyl)-α-d-allopyranoside (***24***)

The substrate **22** (100 mg, 0.28 mmol) was stirred in dry DCM:pyridine (1:1, 3 mL) for 5 min at room temperature, and then mesyl chloride (98 mg, 0.85 mmol) was added dropwise. The reaction was stirred under argon for 4 h at room temperature, then quenched with methanol. The solvent was removed under reduced pressure and diluted with DCM. The organic phases were washed with water and brine and dried over anhydrous MgSO_4_. The crude was purified by column chromatography (hexane-EtOAc 1:3) to give the product **24** as a white solid (72 mg, 60%). The ^1^H NMR (300 MHz, CDCl_3_) δ 7.56–7.43 (m, 2H), 7.42–7.32 (m, 3H), 5.58 (s, 1H), 5.46 (t, J 2.8 Hz, 1H), 4.85 (d, J 3.0 Hz, 1H), 4.36 (dd, J 10.3, 5.2 Hz, 1H), 4.20 (td, J 10.0, 5.2 Hz, 1H), 3.73 (t, J 10.3 Hz, 1H),3.66 (dd, J 9.7, 2.7 Hz, 1H), 3.42 (s, 3H), 3.09 (s, 3H), 2.77 (m, 2H), 2.54 (m, 3H), 1.70–1.55 (m, 4H), and 1.51–1.41 (m, 2H). The ^13^C NMR (75 MHz, CDCl_3_) δ 136.5, 128.7, 127.9, 125.7, 101.3, 98.5, 76.8, 74.3, 68.8, 65.0, 57.8, 55.0, 51.3 39.1, 25.6, 25.1, and 23.9. HRMS (ESI-TOF) calcd. for C_20_H_29_NO_7_SNa [M + Na]^+^: 450.1556; found 450.1558.

### 4.21. Methyl 4,6-O-Benzylidene-2-deoxy-2-methanesulfonamido-3-O-methanesulfonyl-α-d-allopyranoside (***25***)

To the stirred solution of amino alcohol **20** (300 mg, 1.06 mmol) in dry pyridine (5 mL) at 0 °C, mesyl chloride (0.24 mL, 3.19 mmol) was added dropwise. The reaction was left stirring for 24 h, before the solvent was evaporated under a vacuum, and was then diluted with dichloromethane. The organic phase was washed with saturated NaHCO_3_, water, and brine, and dried over anhydrous MgSO_4_. The crude was purified by column chromatography (hexane-EtOAc 1:1) to afford the product **25** as a white solid (350 mg, 75%). The ^1^H NMR (300 MHz, CDCl_3_) δ 7.52–7.31 (m, 5H), 5.59 (s, 1H), 5.20 (t, J 3.2 Hz, 1H), 5.13 (d, J 9.7 Hz, 1H), 4.75 (d, J 4.4 Hz, 1H), 4.39 (dd, J 10.6, 5.2 Hz, 1H), 4.13 (td, J 10.1, 5.2 Hz, 1H), 4.00 (dt, J 9.7, 4.0 Hz, 1H) 3.82–3.67 (m, 2H), 3.45 (s, 3H), 3.08 (s, 3H), and 2.97 (s, 3H). The ^13^C NMR (75 MHz, CDCl_3_) δ 136.7, 129.5, 128.5, 126.0, 102.0, 98.9, 77.5, 76.1, 69.1, 57.6, 56.5, 51.9, 43.6, and 39.3. HRMS (ESI-TOF) calcd. for C_16_H_23_NO_9_S_2_Na [M + Na]^+^: 460.07064; found 460.07192. 

### 4.22. Methyl 4,6-O-Benzylidene-2,3-dideoxy-2-methanesulfonamido-3-thioacetyl-α-d-glucopyranoside (***26***)

The mesylate substrate **25** (350 mg, 0.822 mmol) was dissolved in DMPU (8 mL) and treated with potassium thioacetate (469 mg, 4.11 mmol) at 100 °C for 6 h. The suspension was cooled and diluted with 1:1 EtOAc-Et_2_O (100 mL) and washed with saturated NaHCO_3_ solution (3 × 40 mL). The organic phase was washed with water and brine, and dried over anhydrous MgSO_4_. The crude was purified by column chromatography (hexane-EtOAc 1:1) to afford the inseparable mixture of the thioacetyl product **26** and the elimination adduct (180 mg, 60% product). The pure thioacetyl compound **26** was obtained by following this method: to the stirred a solution of thiol **27** (10 mg, 0.02 mmol) in dichloromethane (1 mL), triethylamine (0.01 mL, 0.06 mmol) and acetic anhydride (0.01 mL, 0.09 mmol) were added dropwise at 0 °C and stirred for 30 min. The solvent was then evaporated, and the crude was directly purified by column chromatography (hexane-EtOAc 1:1) to afford pure thioacetyl **26** (15 mg, 75%) as a white solid. The ^1^H NMR (300 MHz, CDCl_3_) δ 7.52–7.43 (m, 2H), 7.43–7.30 (m, 3H), 5.50 (s, 1H), 4.90 (d, J 10.1 Hz, 1H), 4.82 (d, J 3.5 Hz, 1H), 4.28 (dd, J 10.3, 4.7 Hz, 1H), 3.95 (t, J 11.4 Hz, 1H), 3.93–3.84 (m, 1H), 3.71 (t, J 10.3 Hz, 1H), 3.65 (ddd, J 11.7, 10.1, 3.5 Hz, 1H), 3.54 (dd, J 11.2, 9.0 Hz, 1H), 3.47 (s, 3H), 2.99 (s, 3H), and 2.37 (s, 3H). The ^13^C NMR (75 MHz, CDCl_3_) δ 195.0, 137.0, 129.2, 128.4, 126.3, 102.0, 99.7, 78.0, 69.0, 64.5, 57.5, 55.7, 45.3, 42.4, and 31.0. HRMS (ESI-TOF) calcd. for C_17_H_23_NO_7_S_2_Na [M + Na]^+^: 440.08082; found 440.08197.

### 4.23. Methyl 4,6-O-Benzylidene-2,3-dideoxy-2-methanesulfonamido-3-thio-α-d-glucopyranoside (***27***)

Lithium aluminum hydride (76 mg, 2.01 mmol) was stirred in dry THF (5 mL) at 0 °C, and then the mixture of thioacetate substrate **26** and the elimination by-product (220 mg, 0.287 mmol) in dry THF (5 mL) was added. The reaction was stirred at 0 °C for 30 min, then continued at room temperature for 4 h. After completion of the reaction, the reaction was worked up according to Fieser’s workup: diluted with diethyl ether, followed by water, 15% aq. NaOH solution, water again, then dried over anhydrous MgSO_4_. The organic solvent was evaporated under reduced pressure, and the crude was purified by column chromatography (hexane: EtOAc 7:3, then with 3:2), yielding **27** as a white solid (70 mg, 65%). The ^1^H NMR (300 MHz, CDCl_3_) δ 7.49 (qd, J 4.7, 1.7 Hz, 2H), 7.42–7.33 (m, 3H), 5.55 (s, 1H), 4.86 (d, J 10.3 Hz, 1H), 4.79 (d, J 3.5 Hz, 1H), 4.27 (dd, J 9.3, 3.8 Hz, 1H), 3.82–3.66 (m, 2H), 3.55 (ddd, J 11.1, 10.3, 3.5 Hz, 1H), 3.44 (s, 3H), 3.42–3.27 (m, 2H), 3.13 (s, 3H), and 2.20 (d, J 3.4 Hz, 1H). The ^13^C NMR (75 MHz, CDCl_3_) δ 136.9, 129.3, 128.4, 126.2, 102.0, 99.3, 82.2, 68.9, 63.9, 58.9, 55.7, 42.4, and 41.5. HRMS (ESI-TOF) calcd. for C_17_H_23_NO_7_S_2_Na [M + Na]^+^: 398.07025; found 398.0703.

### 4.24. Methyl 4,6-O-Benzylidene-2-deoxy-2-trifluoromethanesulfonamido-α-d-allopyranoside (***28***) and Methyl 4,6-O-Benzylidene-2-deoxy-2-trifluoromethanesulfonamido-3-O-trifluoromethanesulfonyl-α-d-allopyranoside (***29***)

To the stirred solution of amino alcohol **20** (300 mg, 1 mmol) in dichloromethane (10 mL) at −20 °C, pyridine (0.9 mL, 10 mmol) was added. The reaction was left stirring for 10 min before triflic anhydride (0.53 mL, 3.1 mmol) was added dropwise under argon. After 2 h of reaction, the saturated aqueous solution of NaHCO_3_ was added, the aqueous phase was washed with dichloromethane, and the organic phase was washed with water and brine, and dried over anhydrous MgSO_4_. The crude was purified using column chromatography (hexane-EtOAc 7:3) to afford triflamide **28** (50 mg, 34%), and triflate **29** (200 mg, 51%) as white–yellowish solids. The ^1^H NMR (300 MHz, CDCl_3_) δ 7.52–7.43 (m, 2H), 7.42–7.32 (m, 3H), 5.60 (s, 1H), 4.76 (d, J 4.3 Hz, 1H), 4.38 (dd, J 10.3, 4.9 Hz, 1H), 4.28 (t, J 3.2 Hz, 1H), 4.13 (dd, J 10.1, 4.8 Hz, 1H), 3.82–3.71 (m, 2H), 3.62 (dd, J 9.7, 2.8 Hz, 1H), and 3.47 (s, 3H). The ^13^C NMR (75 MHz, CDCl_3_) δ 136.8, 129.5, 128.5, 126.3, 102.0, 99.3, 78.0, 69.0, 68.7, 57.2, 56.6, and 54.5. The ^19^F NMR (282 MHz, CDCl_3_) δ −78.05. HRMS (ESI-TOF) calcd. for C_15_H_18_F_3_NO_7_S [M−H]^−^: 412.06833; found 412.66700.

To the stirred solution of triflamide **28** (50 mg, 0.12 mmol) in dichloromethane (2.5 mL) at −20 °C, pyridine (0.1 mL, 1.2 mmol) was added. The reaction was left stirring for 10 min before triflic anhydride (0.02 mL, 0.24 mmol) was added dropwise under argon. After 2 h of reaction, the saturated aqueous solution of NaHCO_3_ was added, and the aqueous phase was washed with dichloromethane. The organic phase was washed with water and brine, and dried over anhydrous MgSO_4_. The crude was purified using column chromatography (hexane-EtOAc 7:3) to afford triflate **29** (40 mg, 61%) as a yellow solid. The ^1^H NMR (300 MHz, CDCl_3_) δ 7.52–7.44 (m, 2H), 7.44–7.32 (m, 3H), 5.59 (s, 1H), 5.37 (t, J 2.9 Hz, 1H), 4.78 (d, J 4.3 Hz, 1H), 4.38 (dd, J 10.5, 5.2 Hz, 1H), 4.15 (dd, J 9.9, 5.2 Hz, 1H), 4.01 (t, J 3.9 Hz, 1H), 3.85–3.69 (m, 2H), and 3.49 (s, 3H). The ^13^C NMR (75 MHz, CDCl_3_) δ 135.70, 129.07, 127.91, 125.86, 102.06, 97.07, 81.62, 74.51, 68.27, 57.22, 56.04, and 52.76. The ^19^F NMR (282 MHz, CDCl_3_) δ −73.75 and −77.80. HRMS (ESI-TOF) calcd. for C_16_H_17_F_6_NO_9_S_2_ [M−H]^−^: 544.01762; found 544.01646.

### 4.25. Methyl 4,6-O-Benzylidene-2,3-dideoxy-3-thioacetyl-2-trifluoromethanesulfonamido-α-d-glucopyranoside (***30***)

To the stirred substrate **29** (92 mg, 0.16 mmol) in dry acetonitrile (3 mL), potassium thioacetate (96 mg, 0.84 mmol) was added under argon. The reaction was stirred at 5 °C to 20 °C for 3 h before the solvent was evaporated and diluted with ethyl acetate. The organic phase was then washed with water and brine, and dried over anhydrous MgSO_4_. The crude was then purified with column chromatography (hexane-EtOAc 8:2) to afford **30** (30 mg, 40%) as a white solid. The ^1^H NMR (300 MHz, CDCl_3_) δ 7.53–7.38 (m, 2H), 7.37 (ddd, J 4.6, 3.2, 2.6 Hz, 3H), 5.51 (s, 1H), 4.79 (d, J 3.4 Hz, 1H), 4.30 (dd, J 10.3, 4.8 Hz, 1H), 4.02 (t, J 11.4 Hz, 1H), 3.91 (td, J 9.7, 4.7 Hz, 1H), 3.79–3.65 (m, 2H), 3.62–3.45 (m, 1H), 3.50 (s, 3H), and 2.39 (s, 3H). The ^13^C NMR (75 MHz, CDCl_3_) δ 196.9, 136.8, 129.3, 128.4, 126.3, 102.1, 99.0, 68.9, 64.7, 59.3, 55.9, 44.7, 30.9, 29.8, and 27.0. The ^19^F NMR (282 MHz, CDCl_3_) δ −77.55. HRMS (ESI-TOF) calcd. for C_17_H_20_F_3_NO_7_S_2_ [M−H]^−^: 470.05605; found 470.05588.

### 4.26. Methyl 4,6-O-Benzylidene-2,3-dideoxy-2-trifluoromethanesulfonamido-3-thio-α-d-glucopyranoside (***31***)

To the stirred substrate **30** (10 mg, 0.02 mmol) in dry methanol (2 mL), sodium methoxide in methanol (0.06 mmol, 0.15 M) was added under argon. The reaction was stirred at room temperature overnight before the solvent was evaporated and diluted with dichloromethane and aqueous solution of NH_4_Cl. The water phase was acidified with 1 M HCl and then extracted with dichloromethane. The organic phase was then washed with water and brine, and dried over anhydrous MgSO_4_. The crude was then purified with column chromatography (hexane-EtOAc 8:2) to afford the thiol **31** (7 mg, 76%) as a white solid. The ^1^H NMR (300 MHz, CDCl_3_) δ 7.56–7.44 (m, 2H), 7.44–7.32 (m, 3H), 5.57 (s, 1H), 4.76 (d, J 3.5 Hz, 1H), 4.29 (dd, J 9.8, 4.3 Hz, 1H), 3.79 (dd, J 8.7, 4.5 Hz, 1H), 3.71 (t, J 9.9 Hz, 1H), 3.67–3.61 (m, 1H), 3.47 (s, 3H), 3.57–3.35 (m, 1H), 3.35 (dd, J 7.5, 3.3 Hz, 1H), and 2.12 (d, J 4.2 Hz, 1H). The ^13^C NMR (75 MHz, CDCl_3_) δ 136.8, 129.4, 128.5, 126.2, 102.1, 98.4, 82.0, 68.8, 64.2, 59.7, 55.8, 41.18, and 29.86. The ^19^F NMR (282 MHz, CDCl_3_) δ −77.22. HRMS (ESI-TOF) calcd. for C_15_H_18_F_3_NO_6_S_2_ [M−H]^−^: 428.04549; found 428.04488.

## Figures and Tables

**Table 1:** Results of the diethylzinc additions to benzaldehyde.

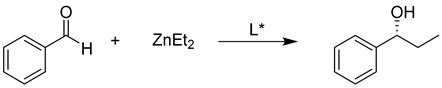						
Entry ^a^	ZnEt_2_ (equiv.)	L* (equiv.)	Solvent	Time [h]	Yield [%]	e.r. (Config.)
1 ^b^	3	**10** (0.1)	DCM	18	85	54.5:45.5 (*S*)
2 ^b^	3	**10** (0.1)	Toluene	18	95	57:43 (*S*)
3	2	**16** (0.1)	Toluene	20	83	55:45 (*R)*
4	2	**10** (0.1)	Toluene	20	87	74.5:25.5 (*S)*
5	2	**31** (0.1)	Toluene	24	82	64:36 (*S*)
6	2	**27** (0.1)	Toluene	24	86	80.5:19.5 (*S*)
7	2	**15** (0.1)	Toluene	20	35	53:47 (*R*)
8	2	**9** (0.1)	Toluene	20	61	71.5:28.5 (*S*)
9	2	**26** (0.1)	Toluene	20	73	54.5:45.5 (*R*)
10	2	**30** (0.1)	Toluene	20	70	51.5:48.5 (*R*)

^a^ All reactions performed at r.t.; ^b^ In the presence of Ti(O*i*Pr)_4_ (1.4 equiv.).

## Data Availability

Raw data are available from the authors.

## Supplementary Materials

The supporting information can be downloaded at: https://www.mdpi.com/article/10.3390/ijms25105542/s1.
