# Supplementary material for: Synthesis of Novel 3-Deoxy-3-thio Derivatives of d-Glucosamine and Their Application as Ligands for the Enantioselective Addition of Diethylzinc to Benzaldehyde
Source: Int J Mol Sci. 2024 May 19;25(10):5542. doi: 10.3390/ijms25105542 (PMC11121937; doi:10.3390/ijms25105542)
Supplement: Supplementary file 1 [file ijms-25-05542-s001.zip › ijms-3009386-supplementary.pdf]

SUPPLEMENTARY MATERIAL

Synthesis of novel 3-deoxy-3-thio derivatives of D-glucosamine and their application as ligands for the enantioselective addition of diethylzinc to benzaldehyde.

Authors

Yusuf Zaim Hakim, Tomasz Bauer*

Faculty of Chemistry, University of Warsaw, L. Pasteura 1, PL-02-093 Warsaw, Poland

* Correspondence: tbauer@chem.uw.edu.pl

Content

1. ^{1}H NMR and ^{13}C NMR spectra of synthesized compounds

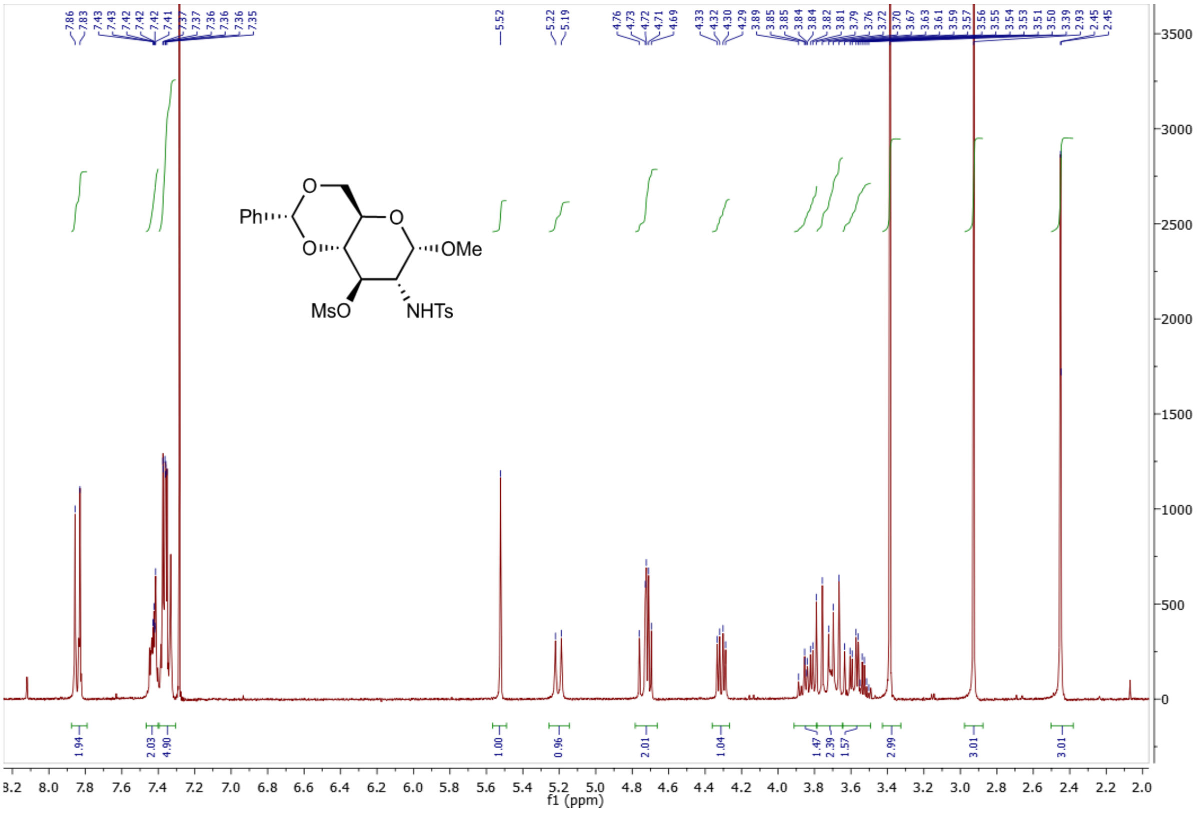

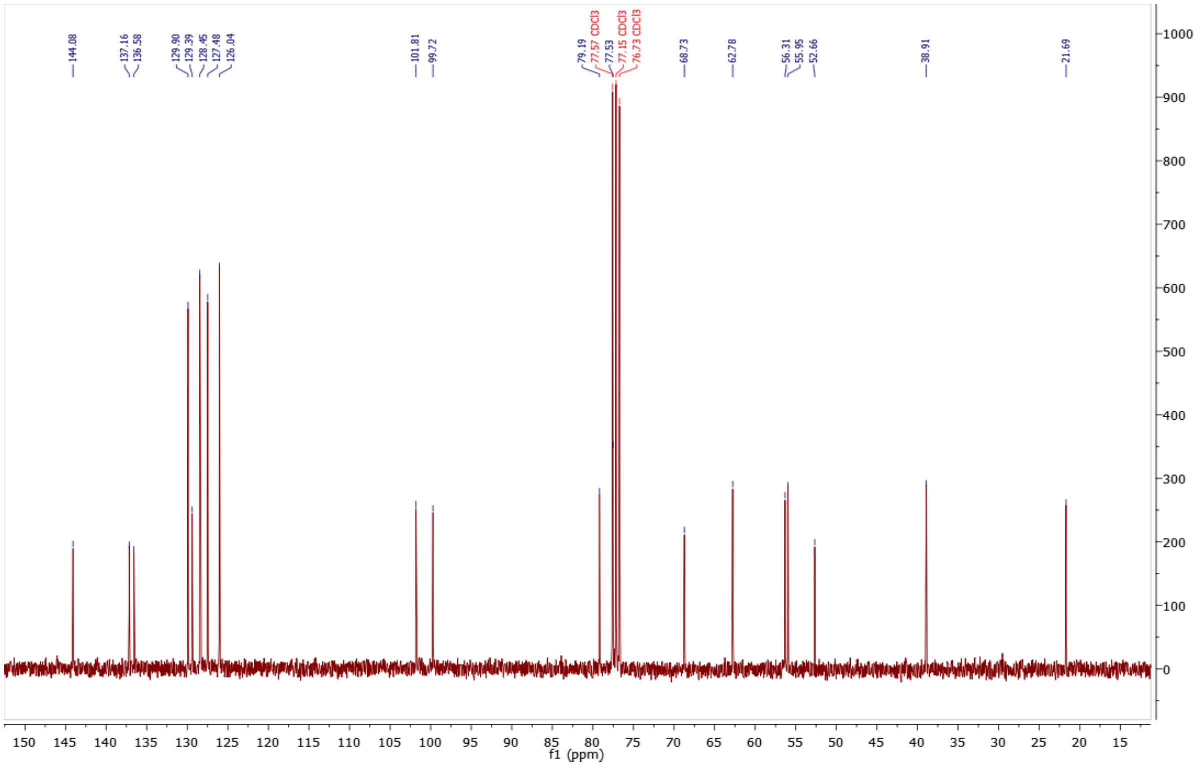

Fig. S1. ^{1}H NMR and ^{13}C NMR Spectrum of Compound **6**

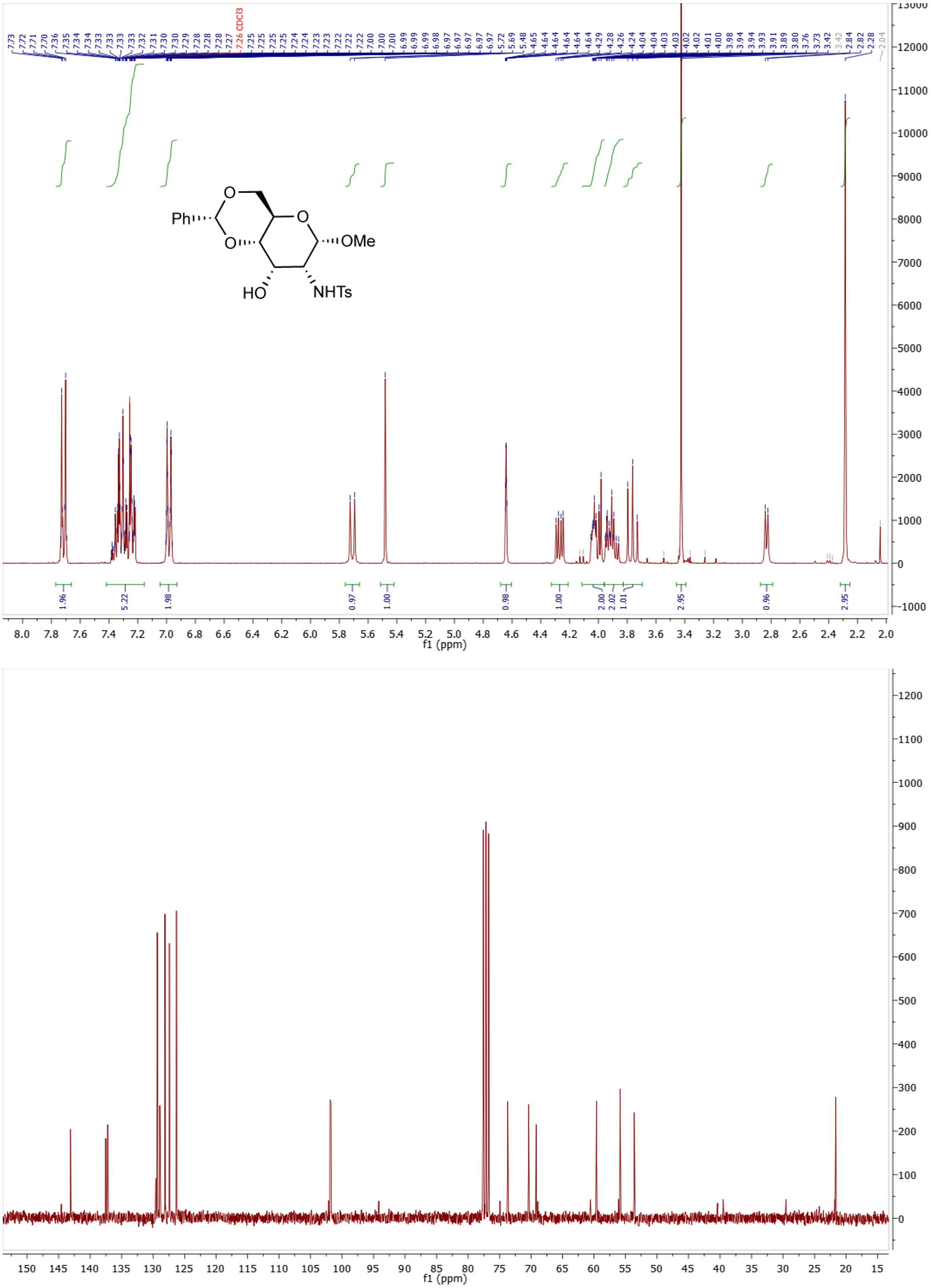

Fig. S2. ^{1}H NMR and ^{13}C NMR Spectrum of Compound **7**

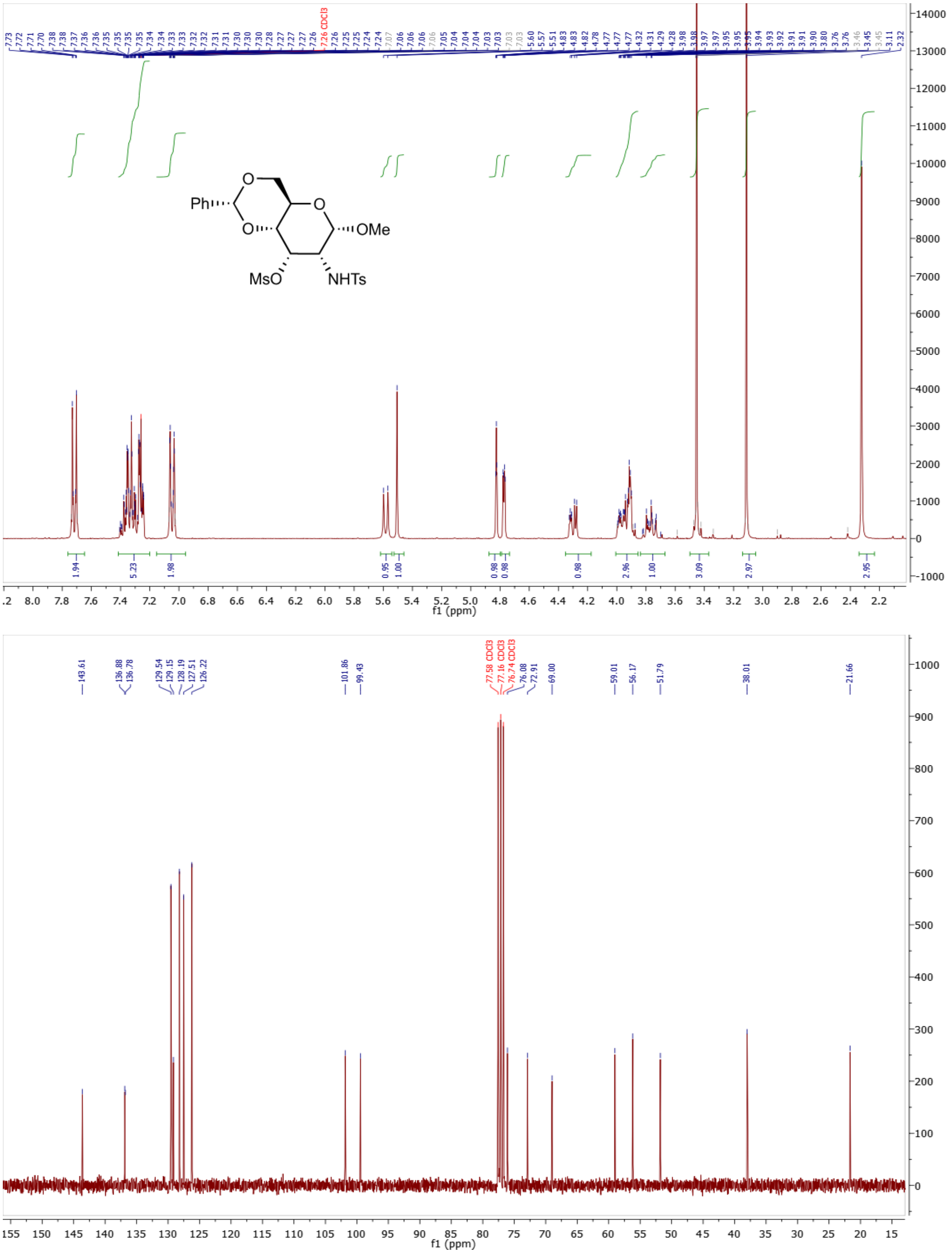

Fig. S3. ^{1}H NMR and ^{13}C NMR Spectrum of Compound **8**

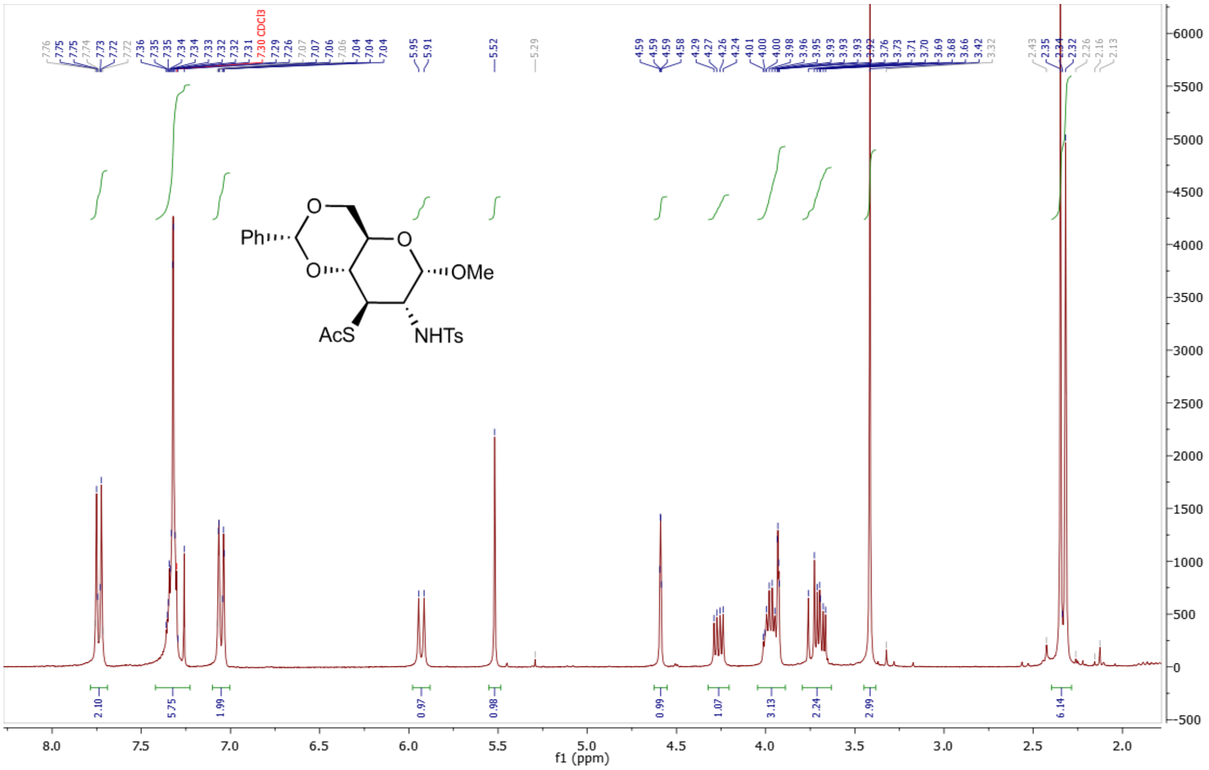

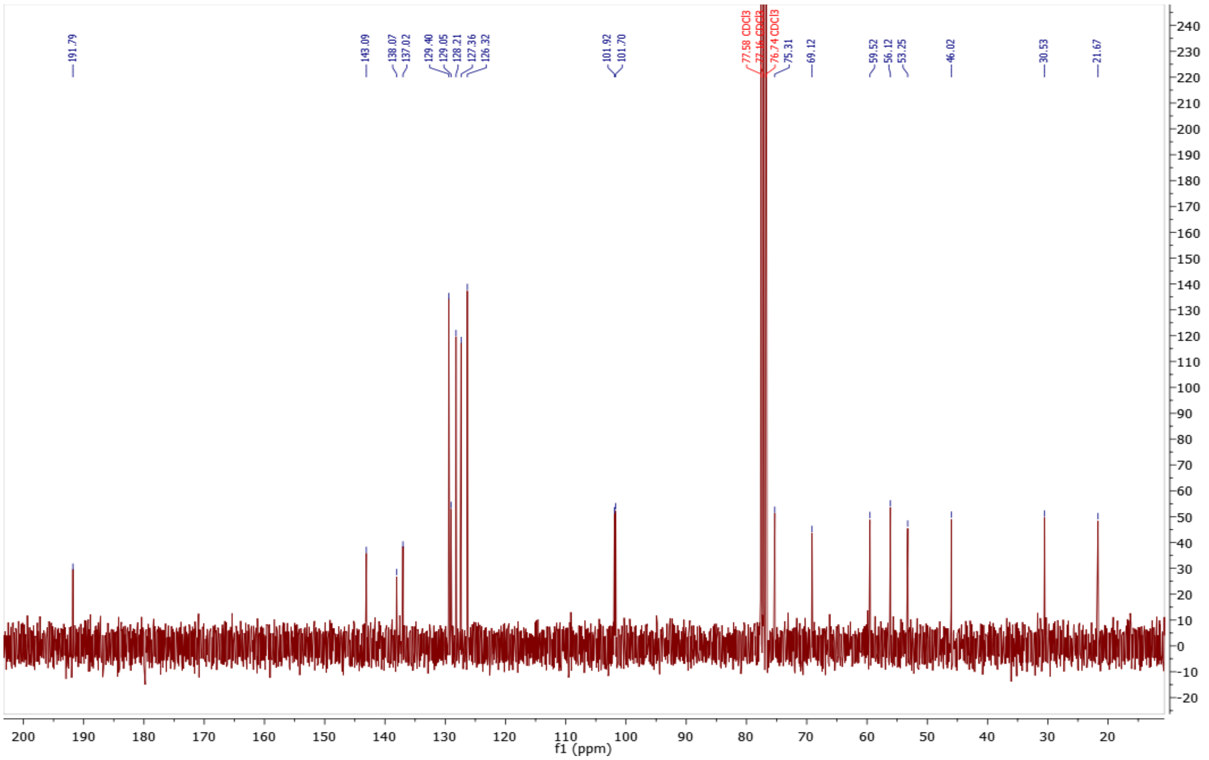

Fig. S4. ^{1}H NMR and ^{13}C NMR Spectrum of Compound **9**

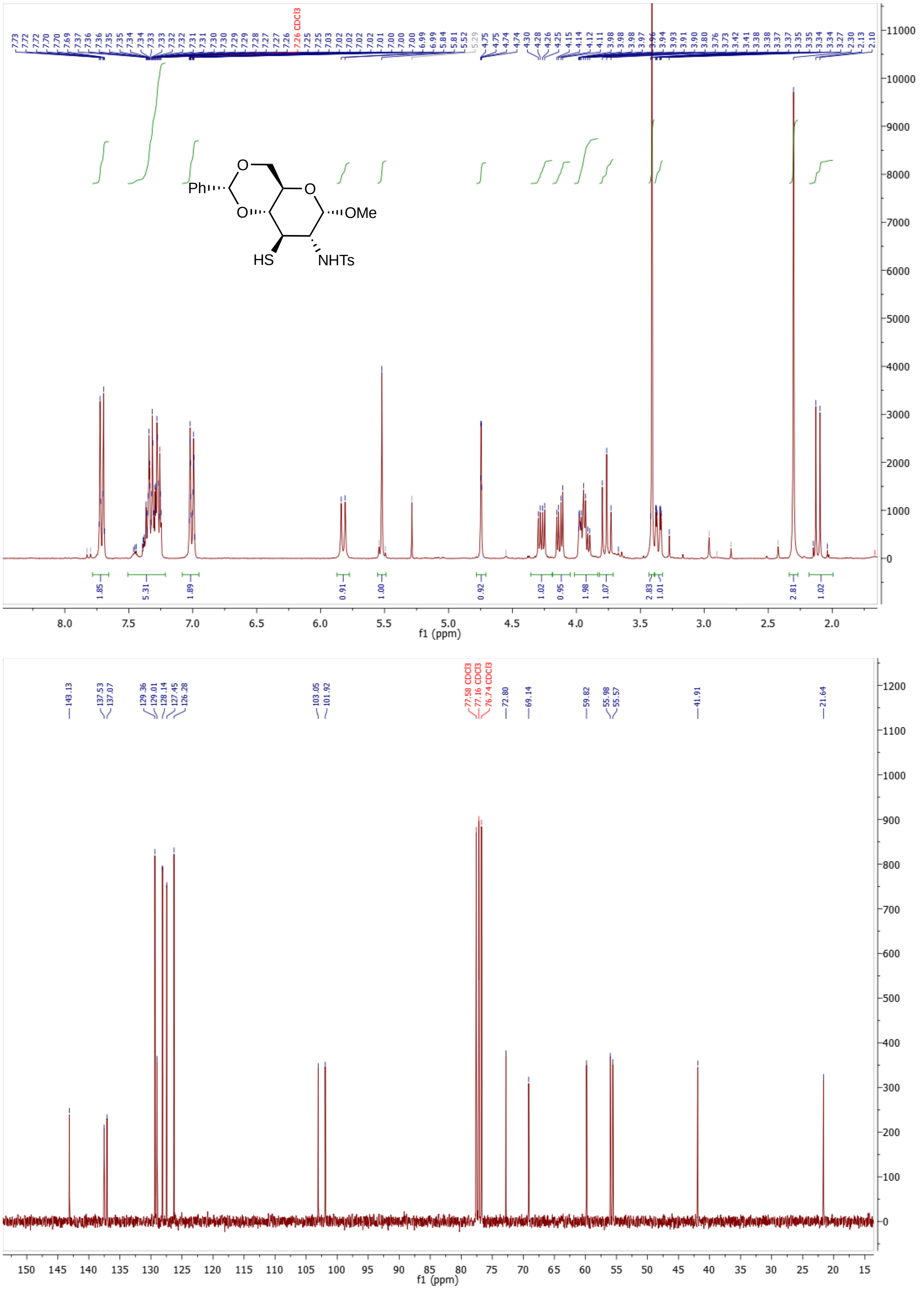

Fig. S5. ^{1}H NMR and ^{13}C NMR Spectrum of Compound **10**

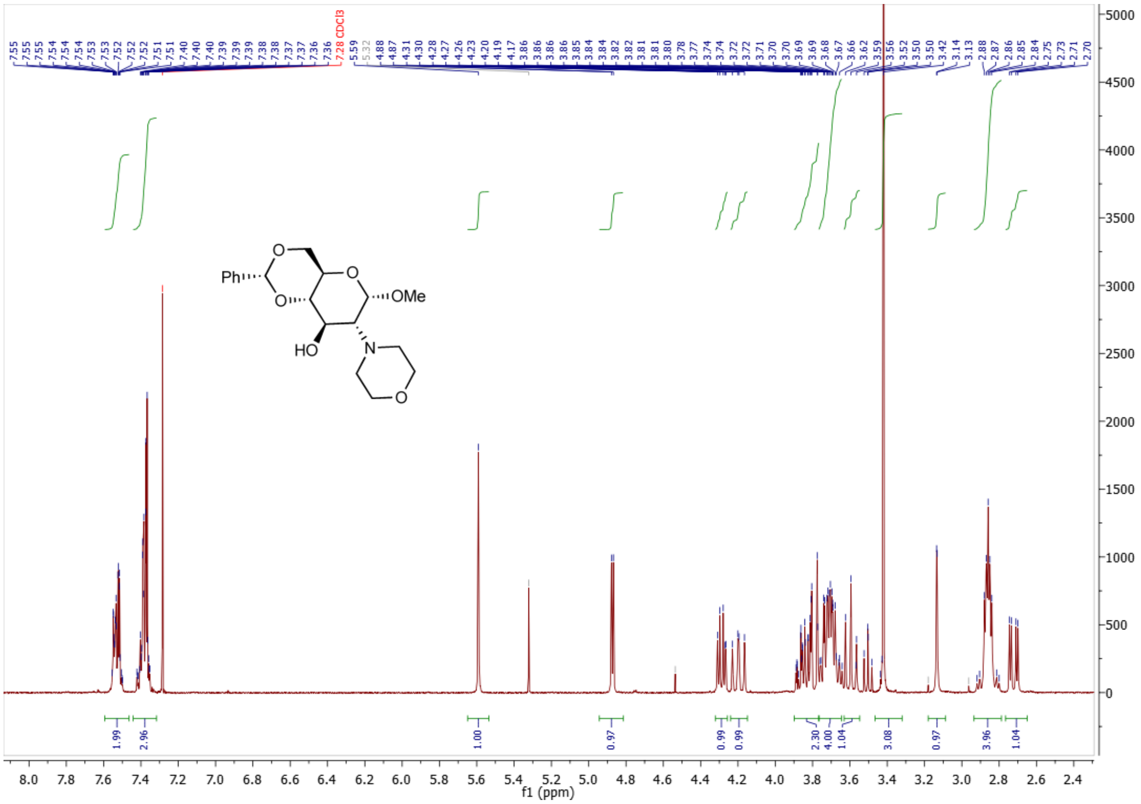

Fig. S6. ^{1}H NMR Spectrum of Compound **11**

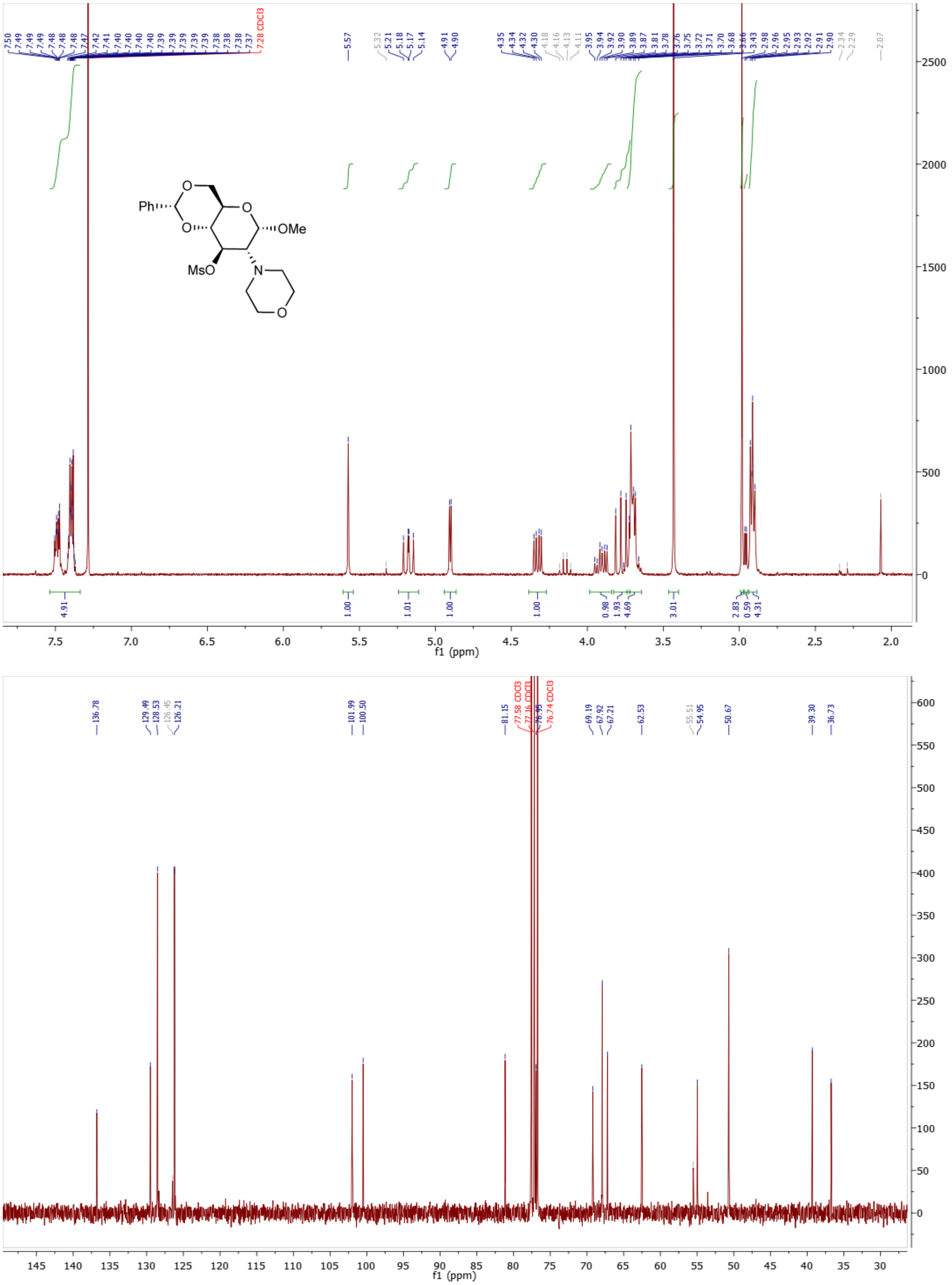

Fig. S7. ^{1}H NMR and ^{13}C NMR Spectrum of Compound **12**

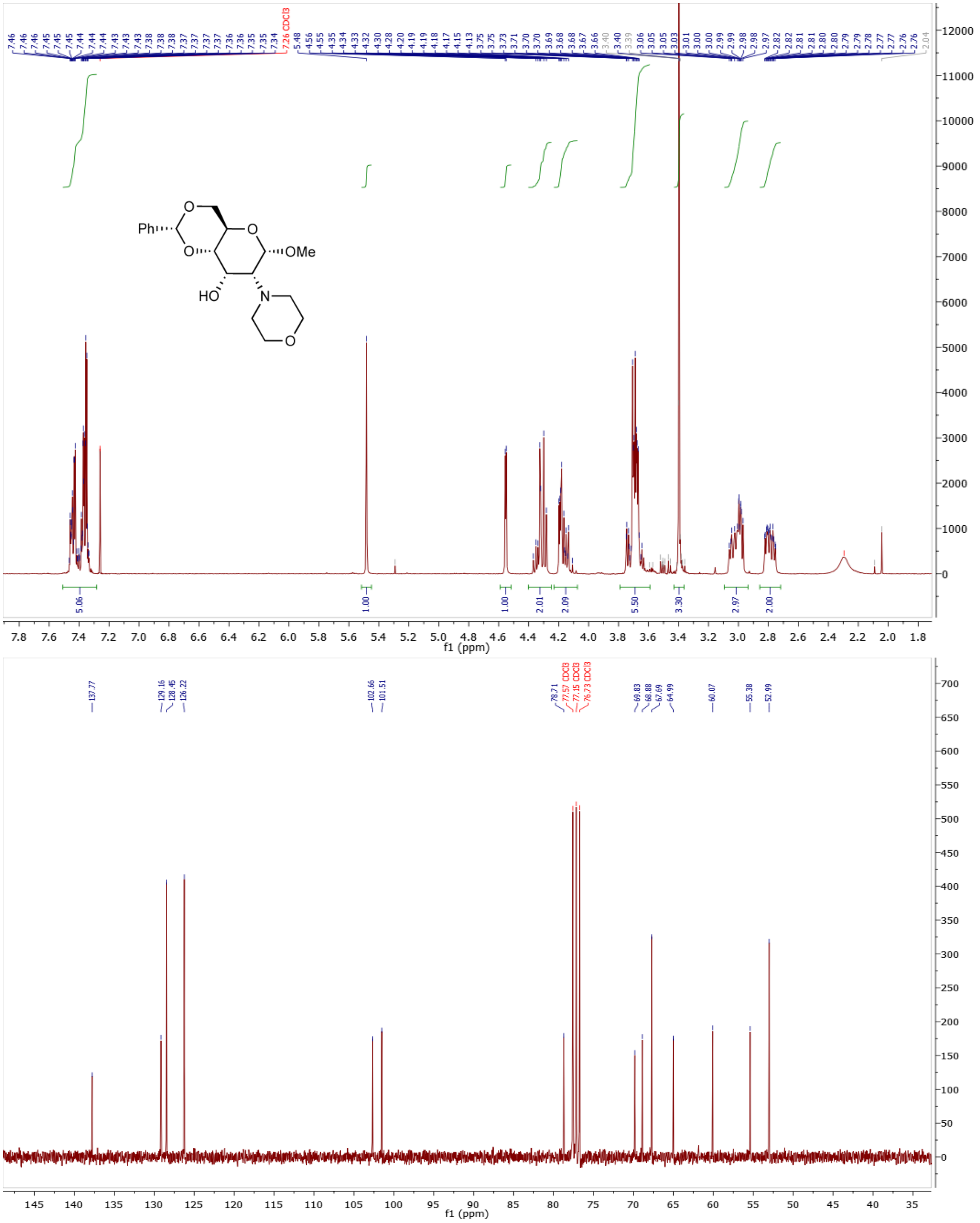

Fig. S8. ^{1}H NMR and ^{13}C NMR Spectrum of Compound **13**

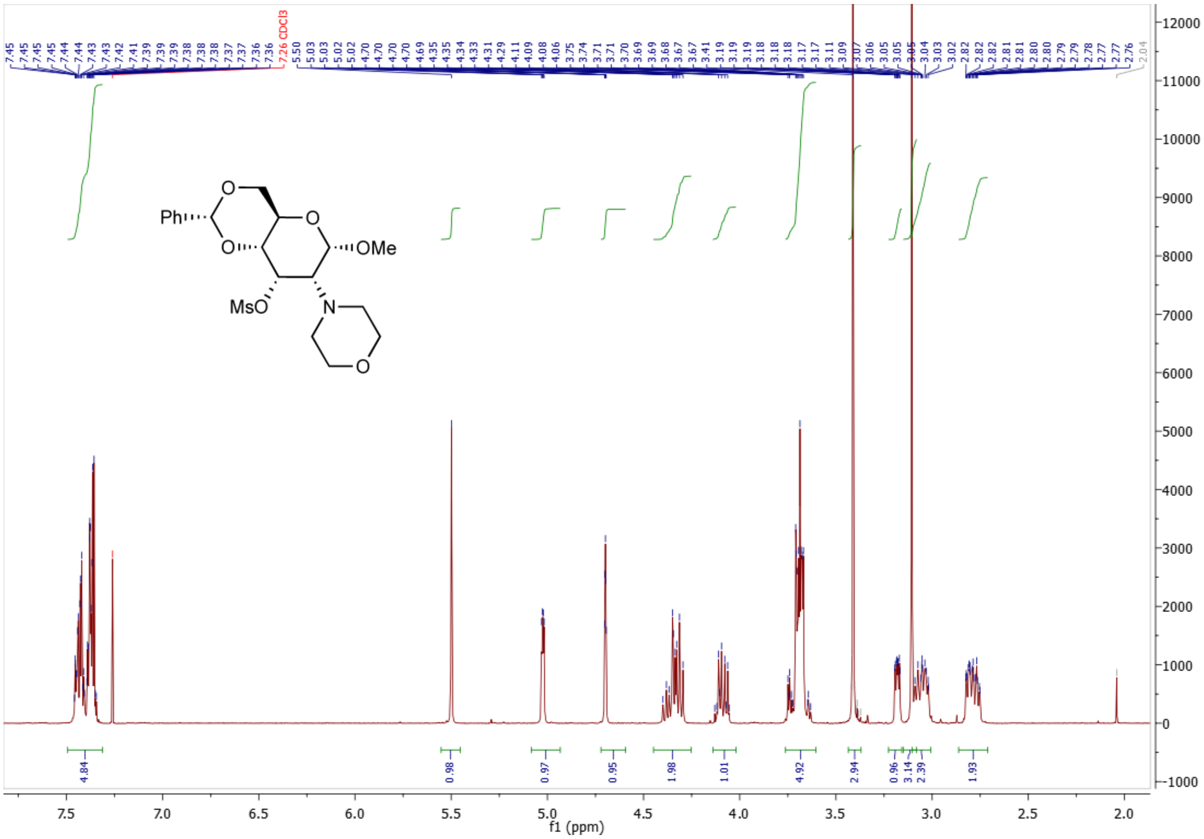

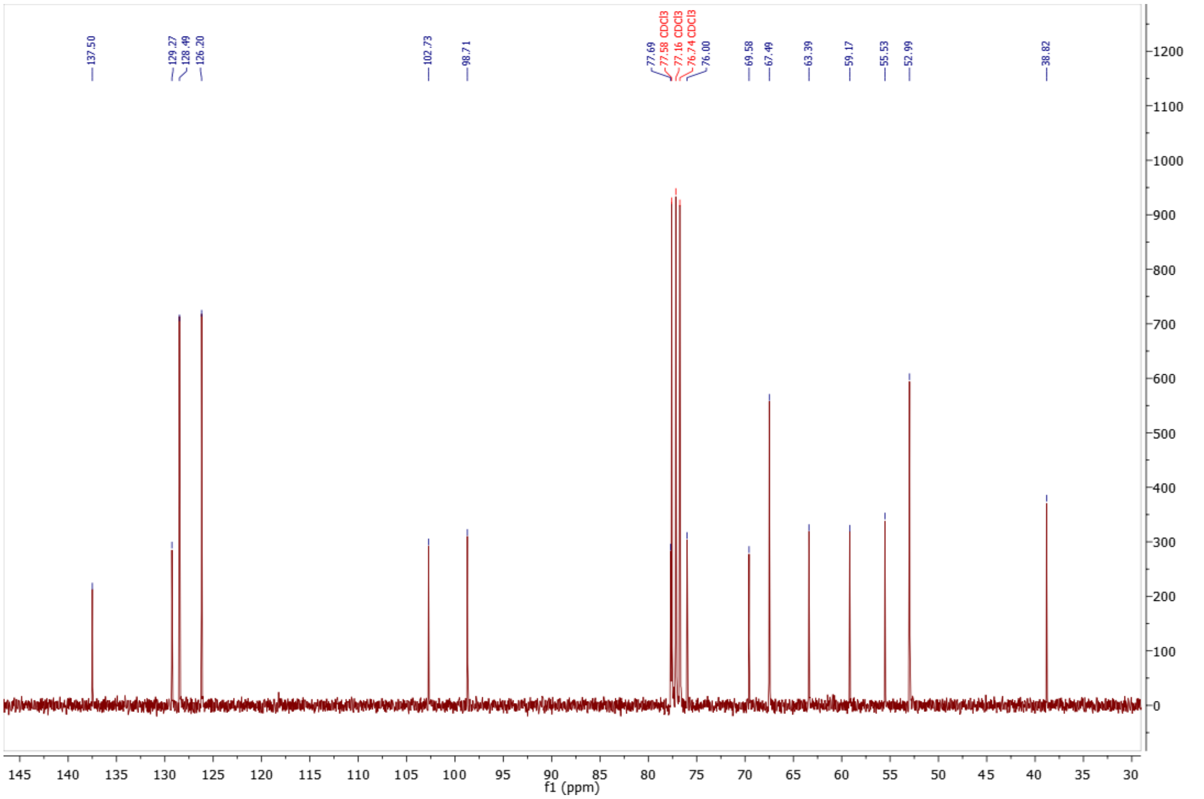

Fig. S9. ^{1}H NMR and ^{13}C NMR Spectrum of Compound **14**

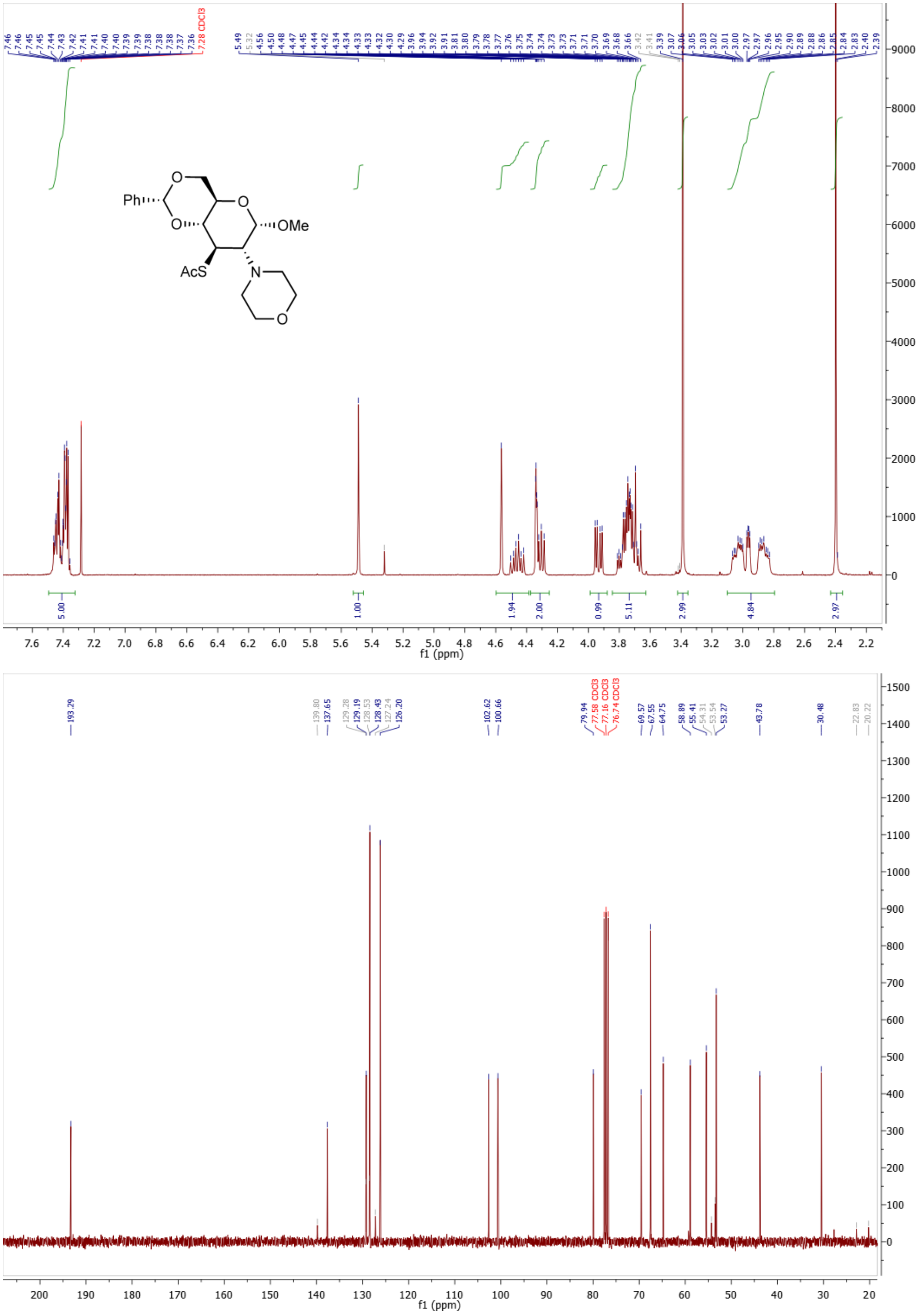

Fig. S10. ^{1}H NMR and ^{13}C NMR Spectrum of Compound **15**

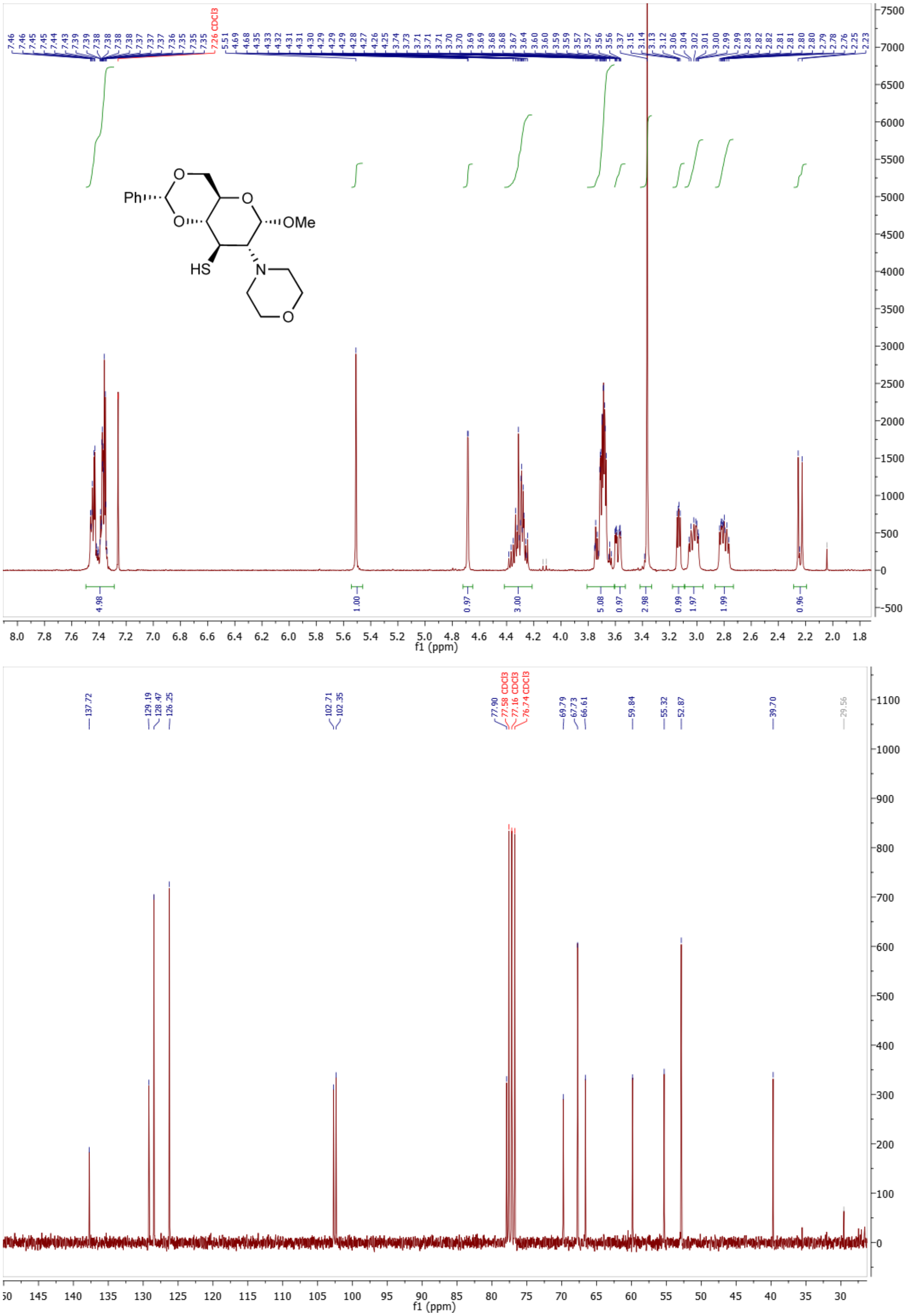

Fig. S11. ^{1}H NMR and ^{13}C NMR Spectrum of Compound **16**

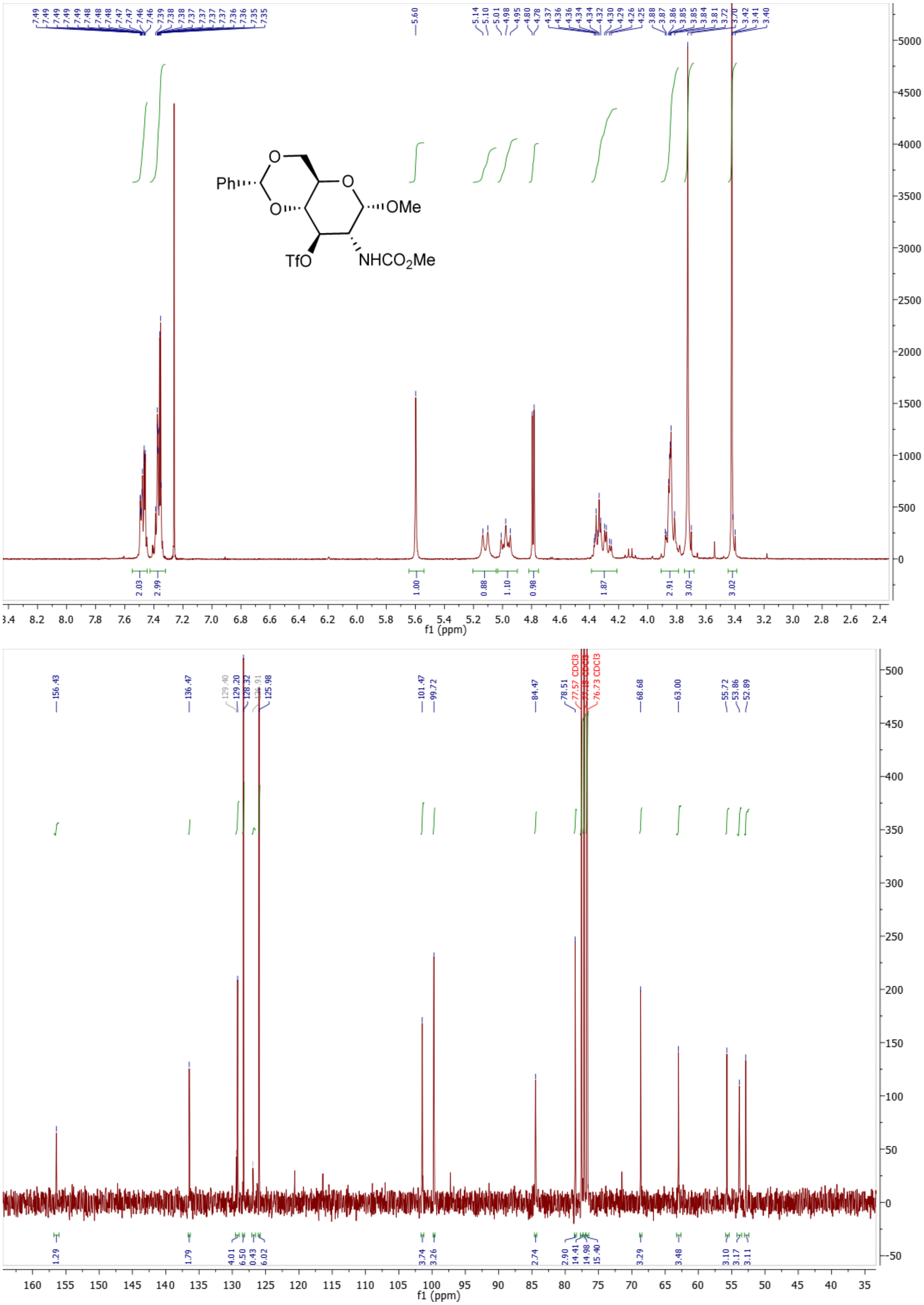

Fig. S12. ^{1}H NMR and ^{13}C NMR Spectrum of Compound **18**

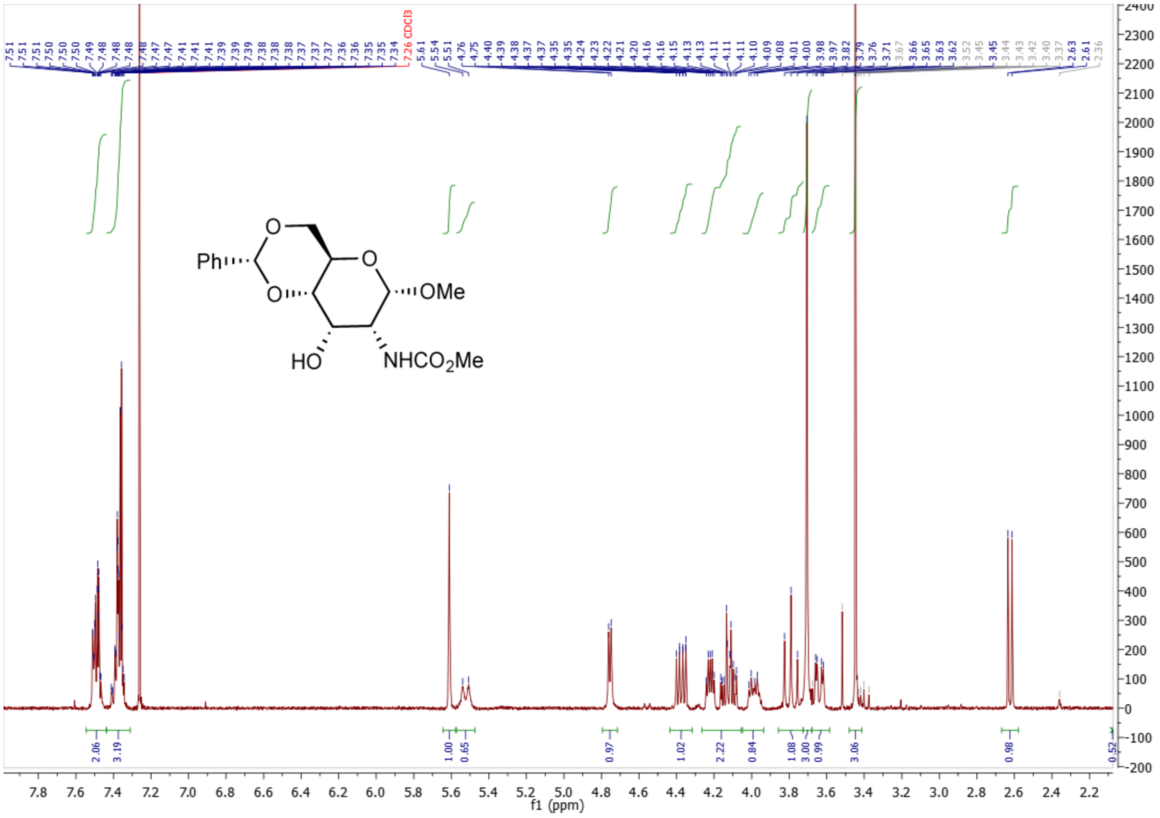

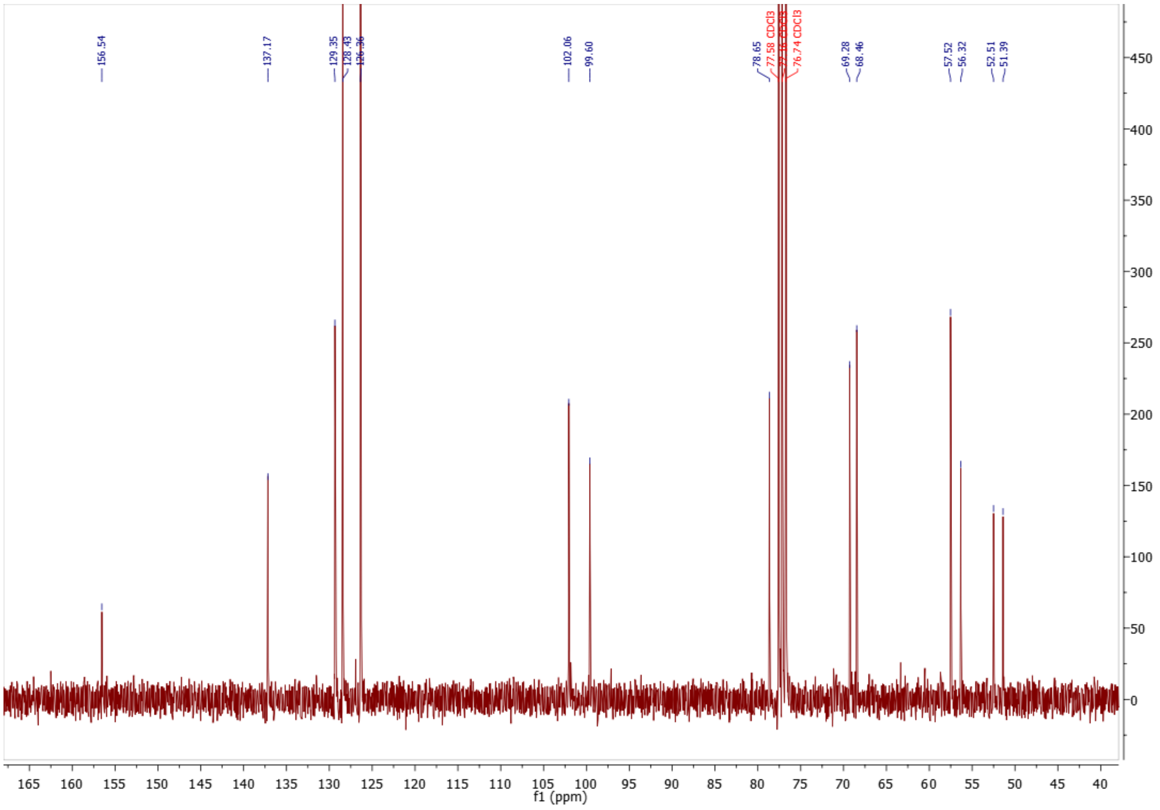

Fig. S13. ^{1}H NMR and ^{13}C NMR Spectrum of Compound **19**

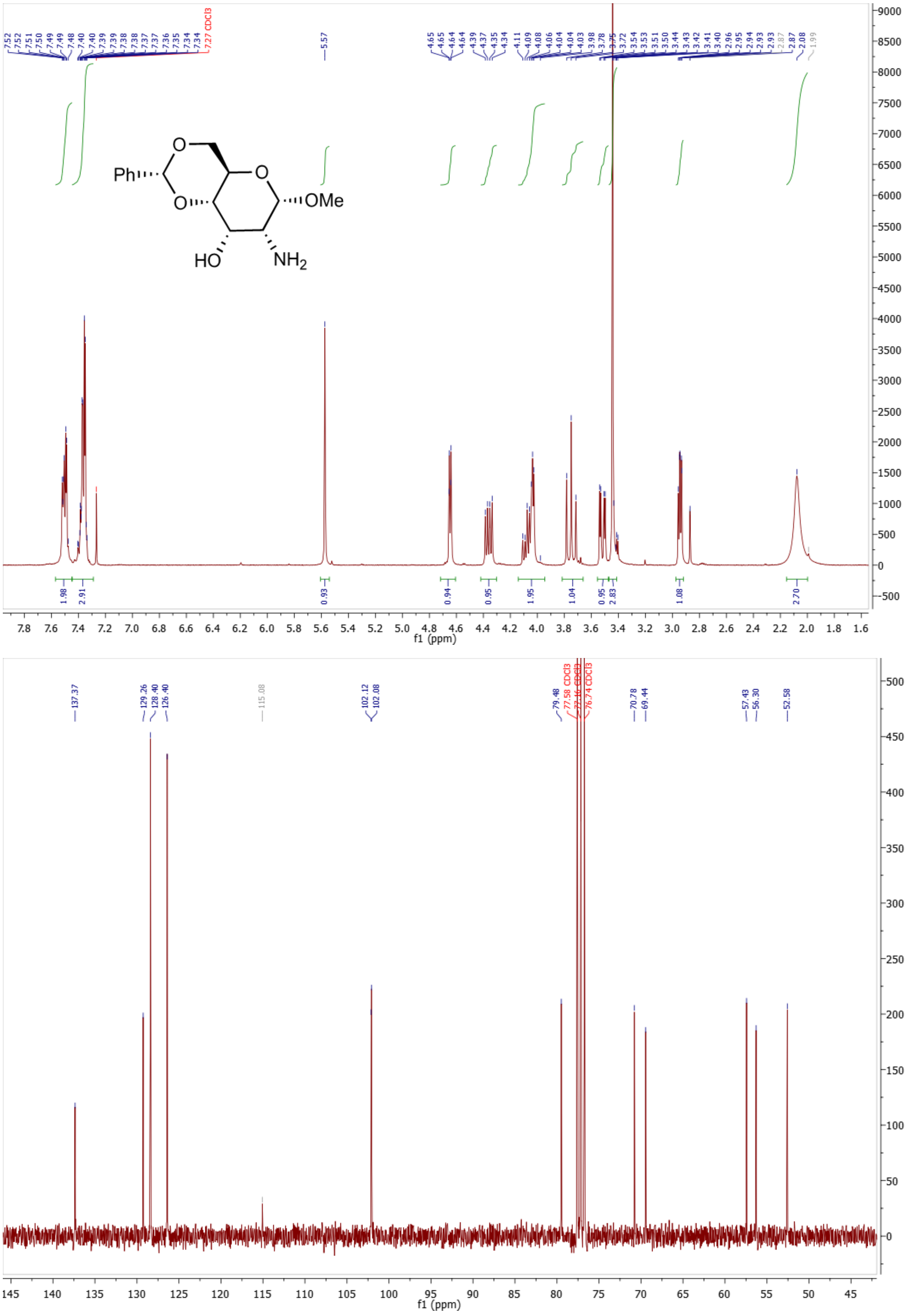

Fig. S14. ^{1}H NMR and ^{13}C NMR Spectrum of Compound **20**

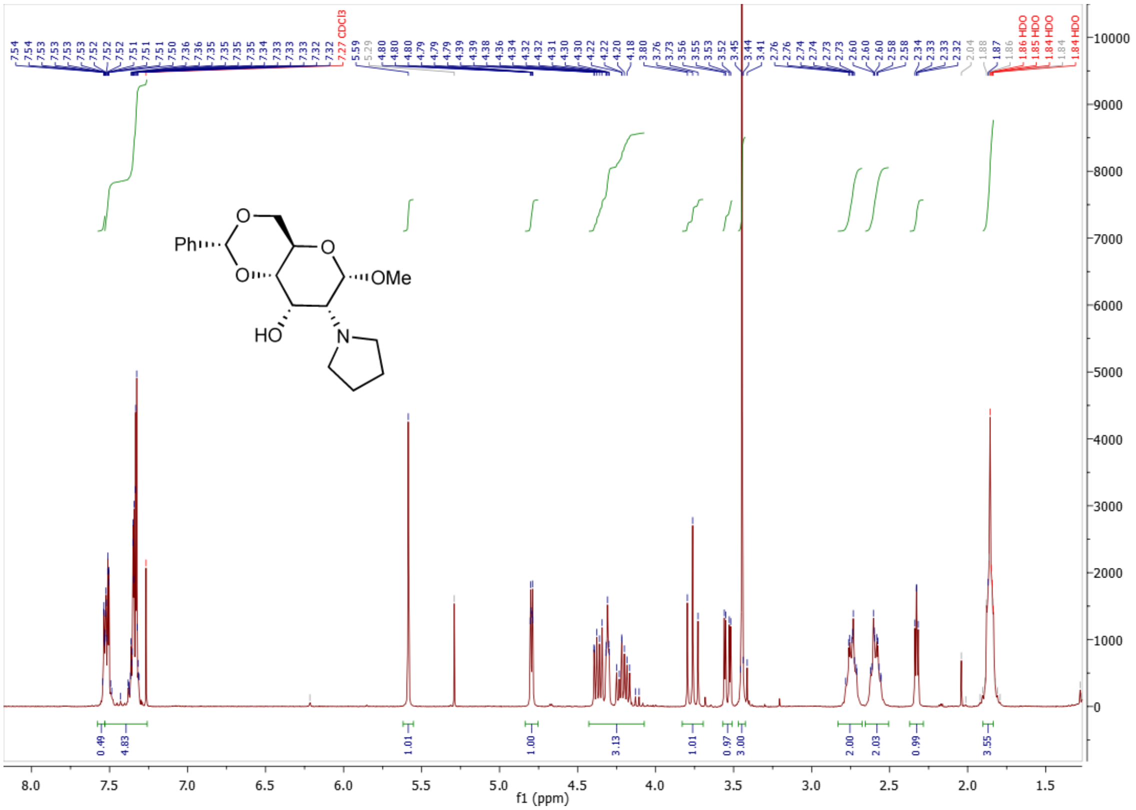

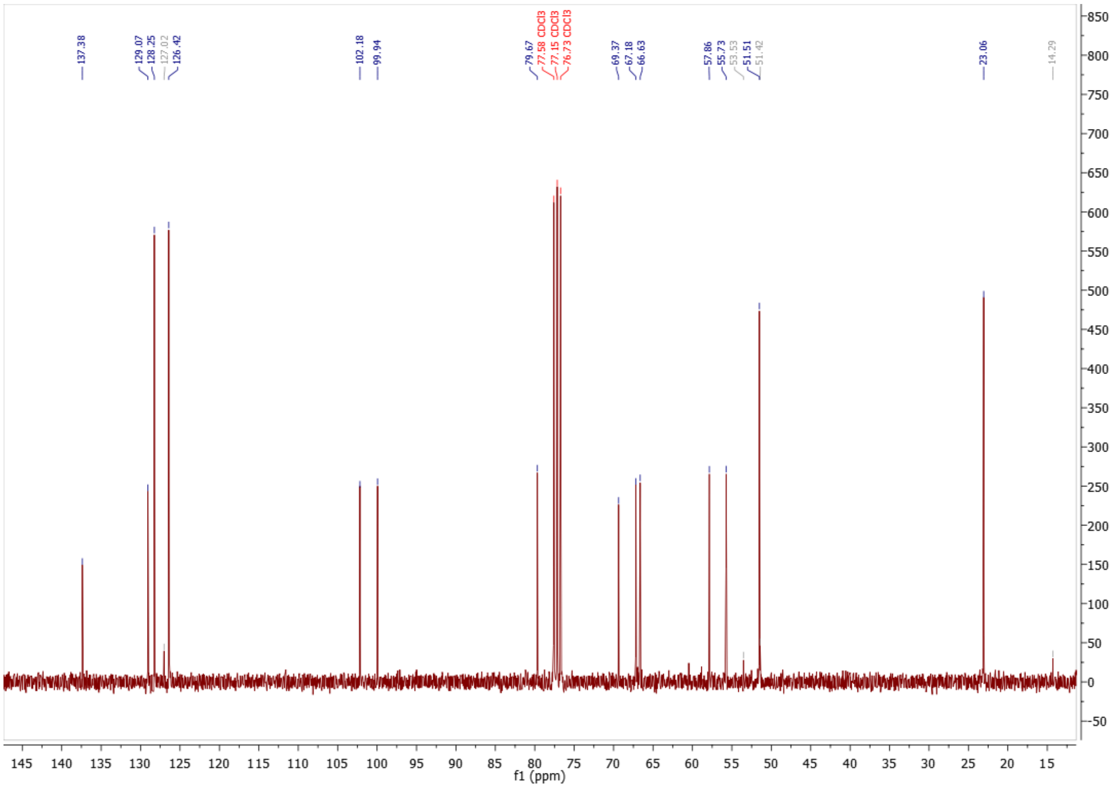

Fig. S15. ^{1}H NMR and ^{13}C NMR Spectrum of Compound **21**

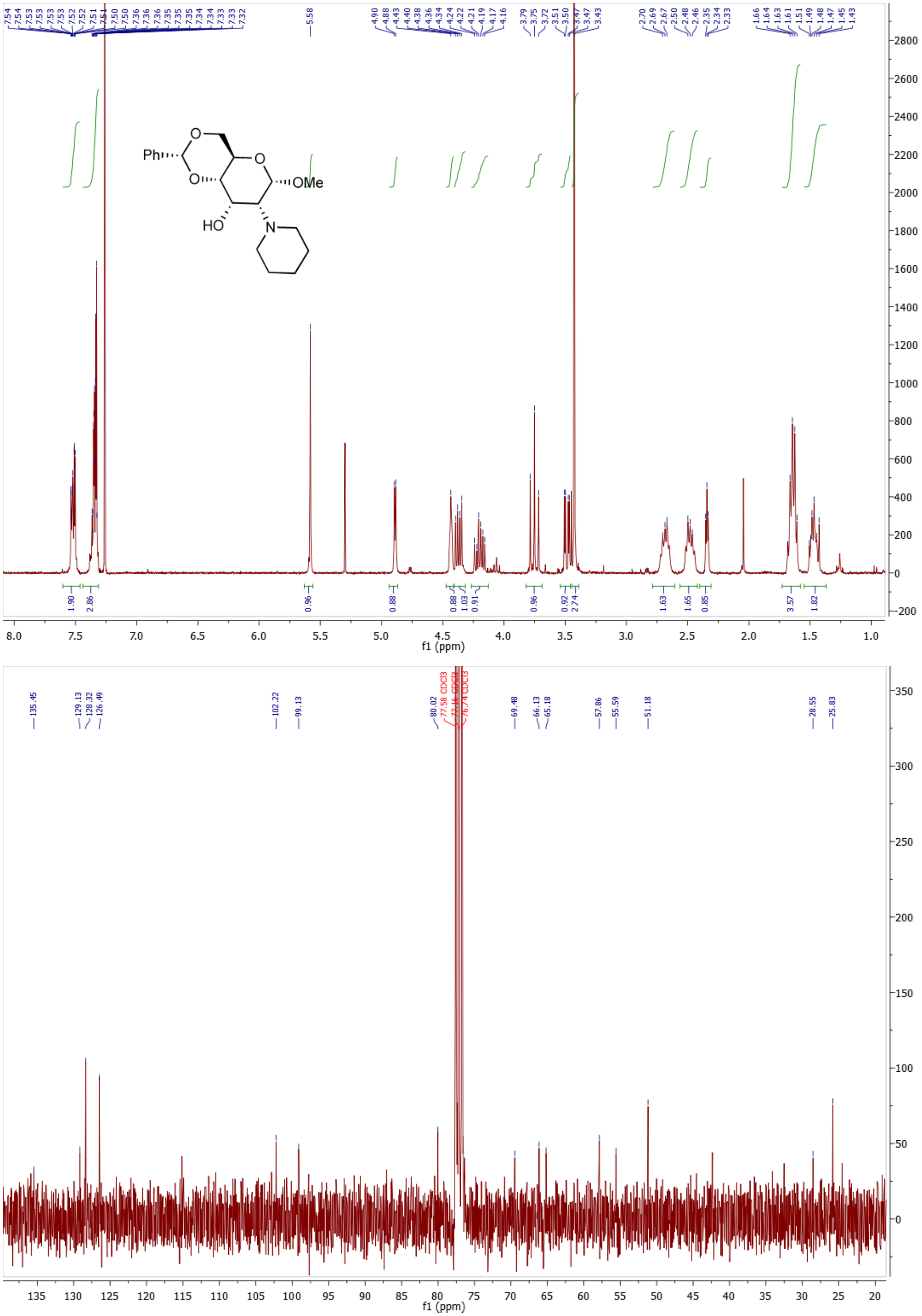

Fig. S16. ^{1}H NMR and ^{13}C NMR Spectrum of Compound **22**

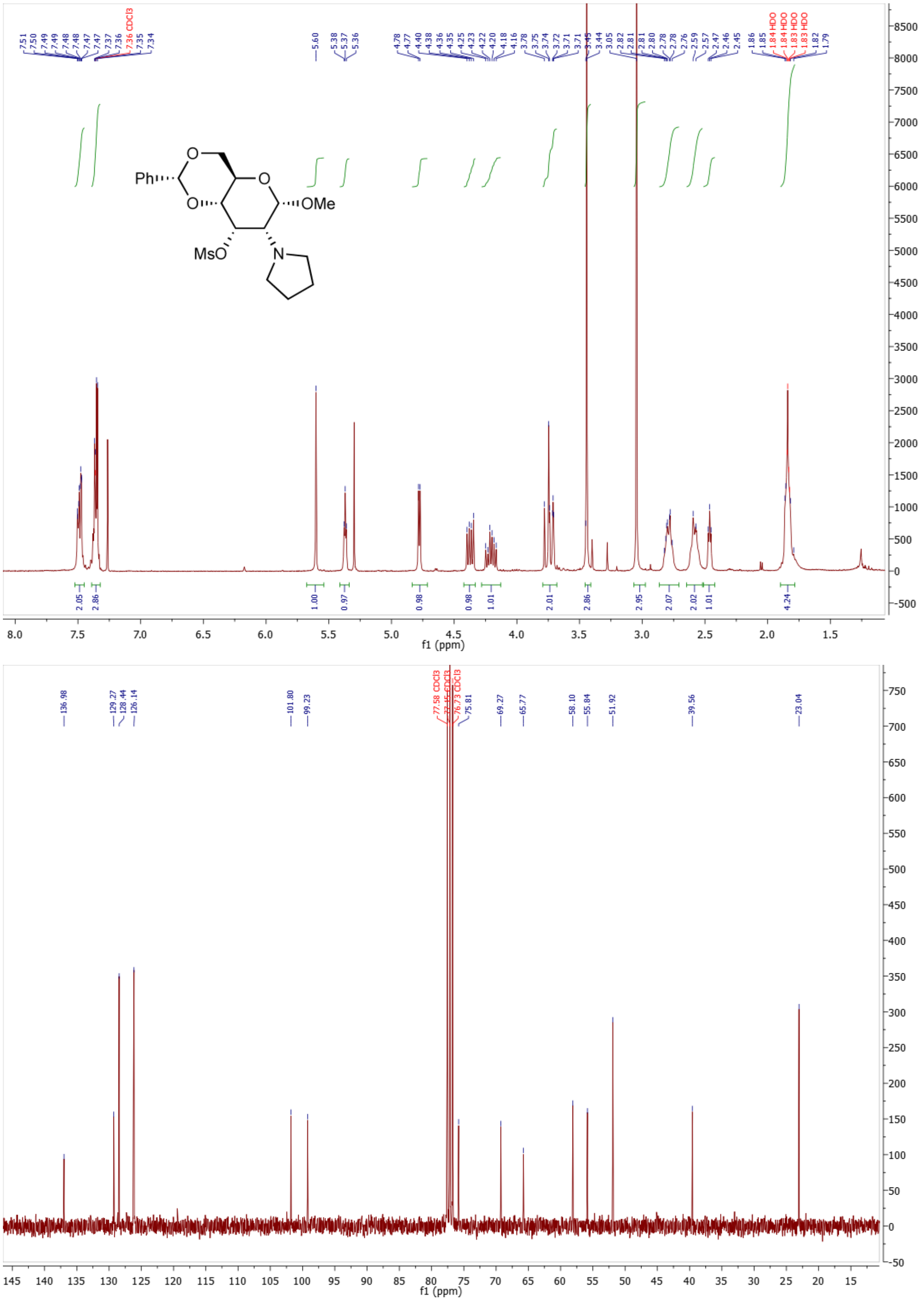

Fig. S17. ^{1}H NMR and ^{13}C NMR Spectrum of Compound **23**

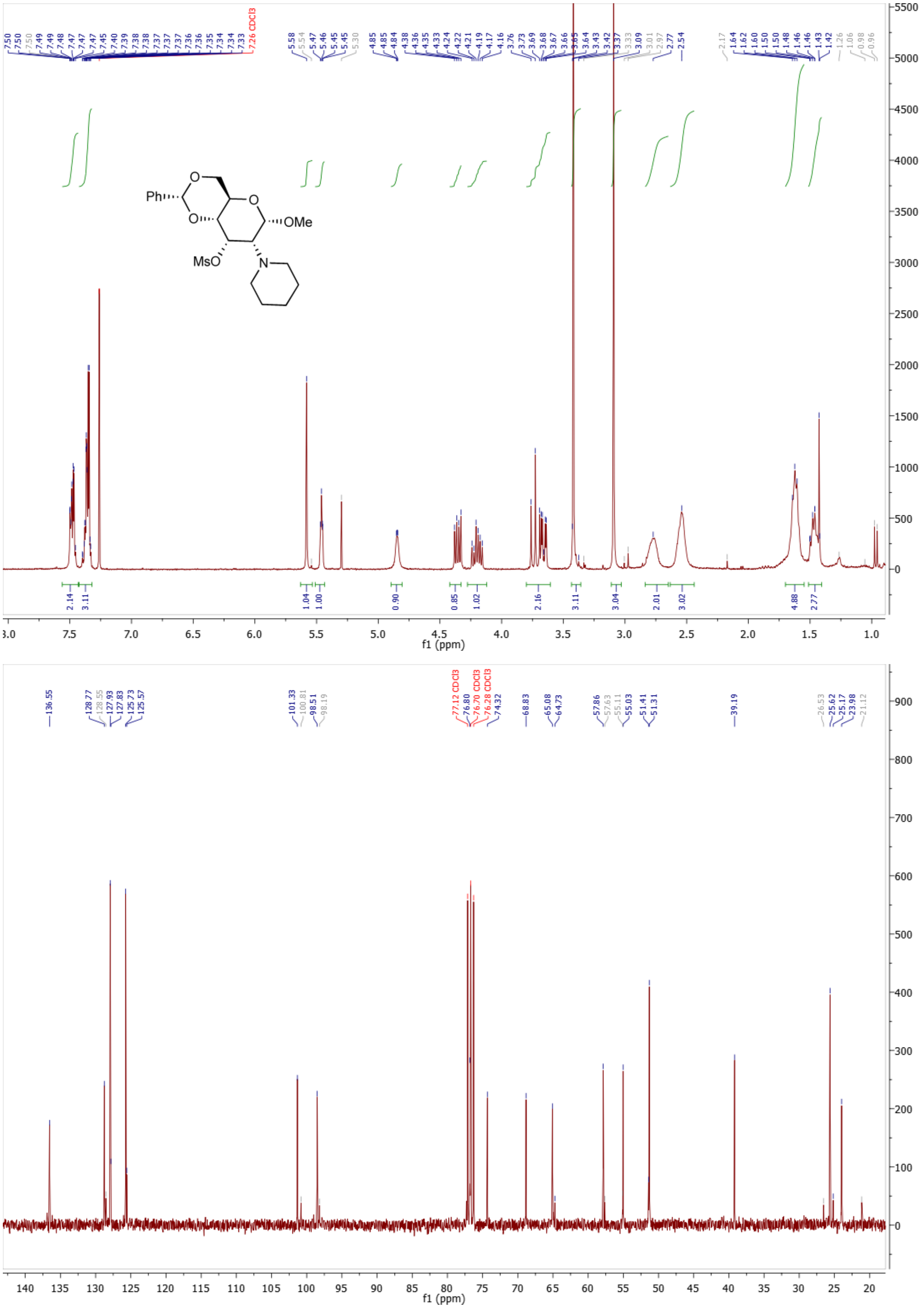

Fig. S18. ^{1}H NMR and ^{13}C NMR Spectrum of Compound **24**

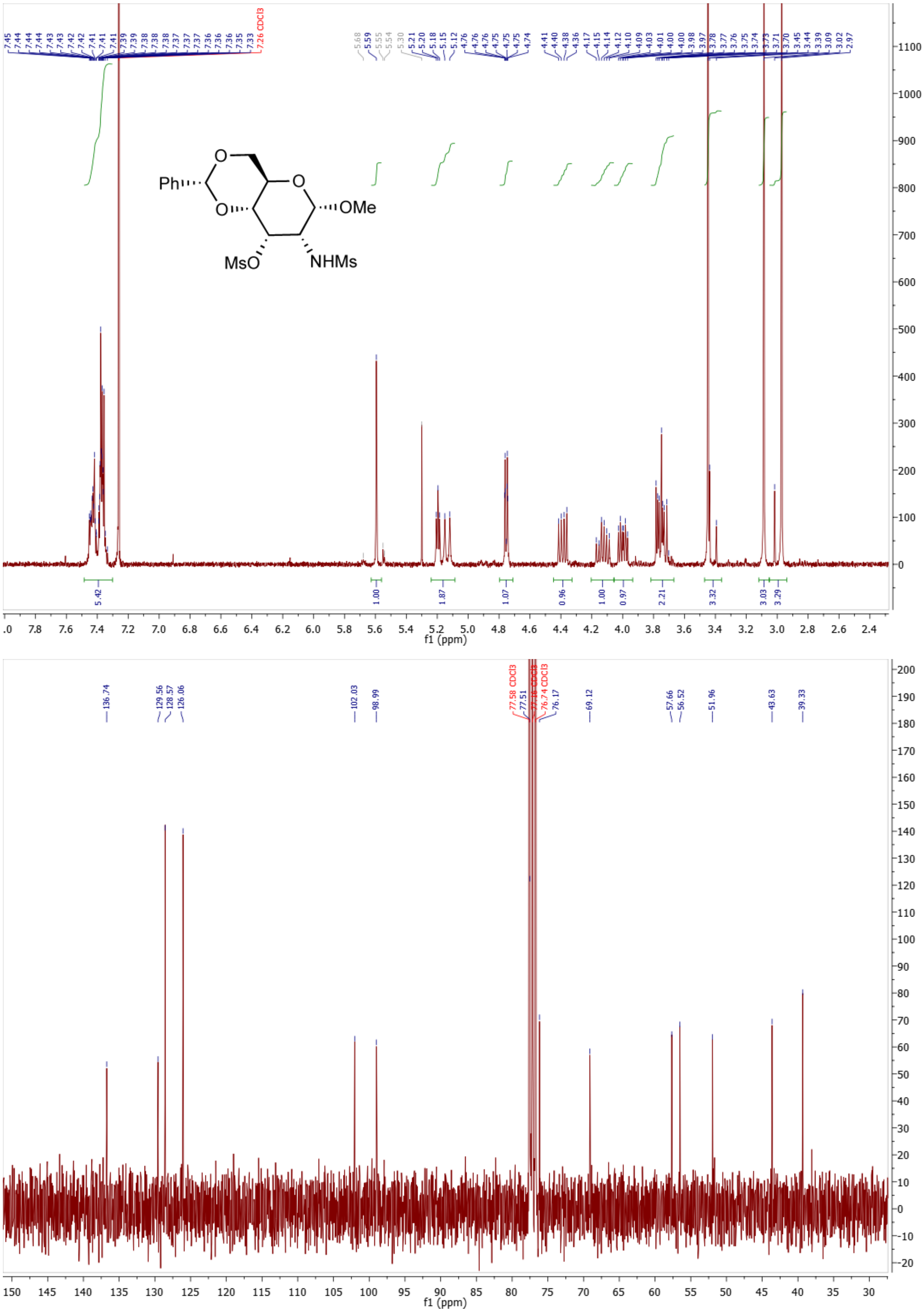

Fig. S19. ^{1}H NMR and ^{13}C NMR Spectrum of Compound **25**

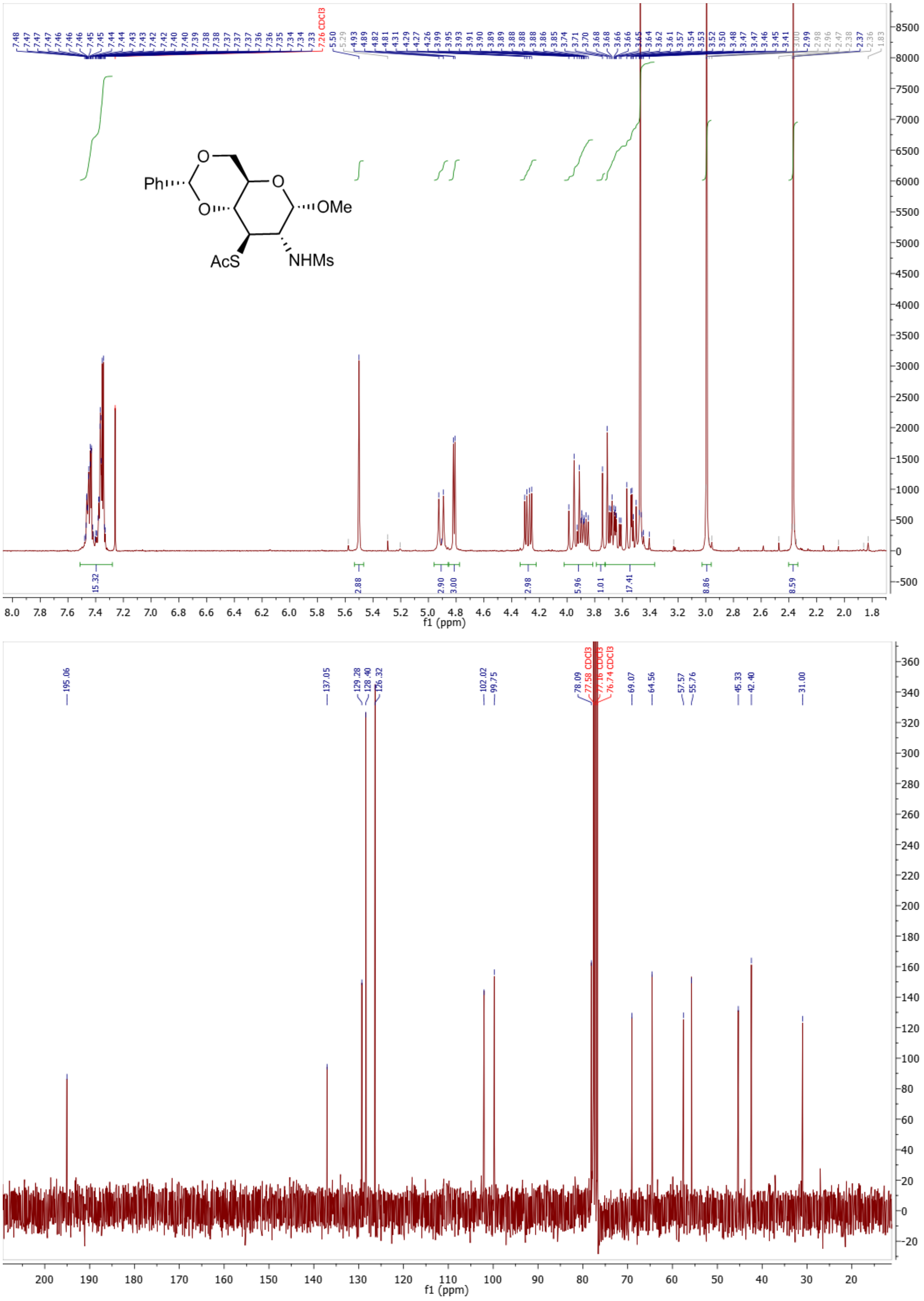

Fig. S20. ^{1}H NMR and ^{13}C NMR Spectrum of Compound **26**

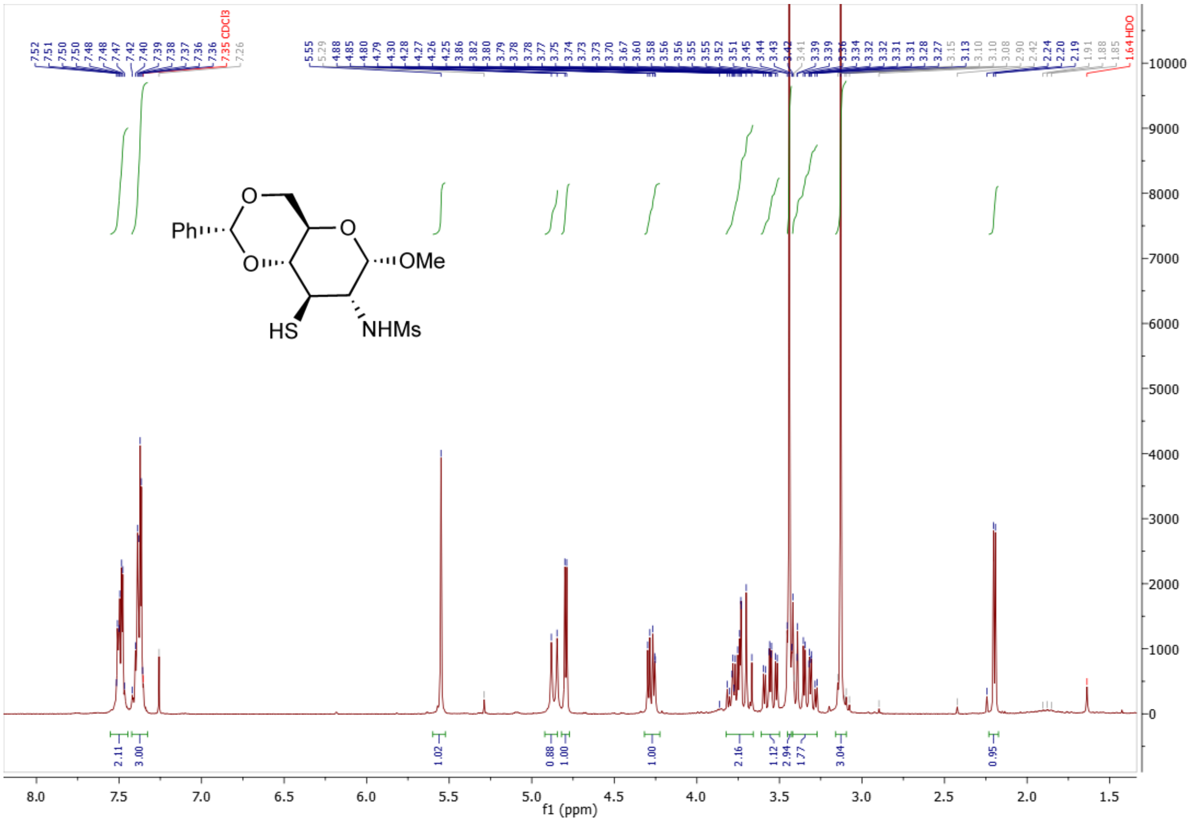

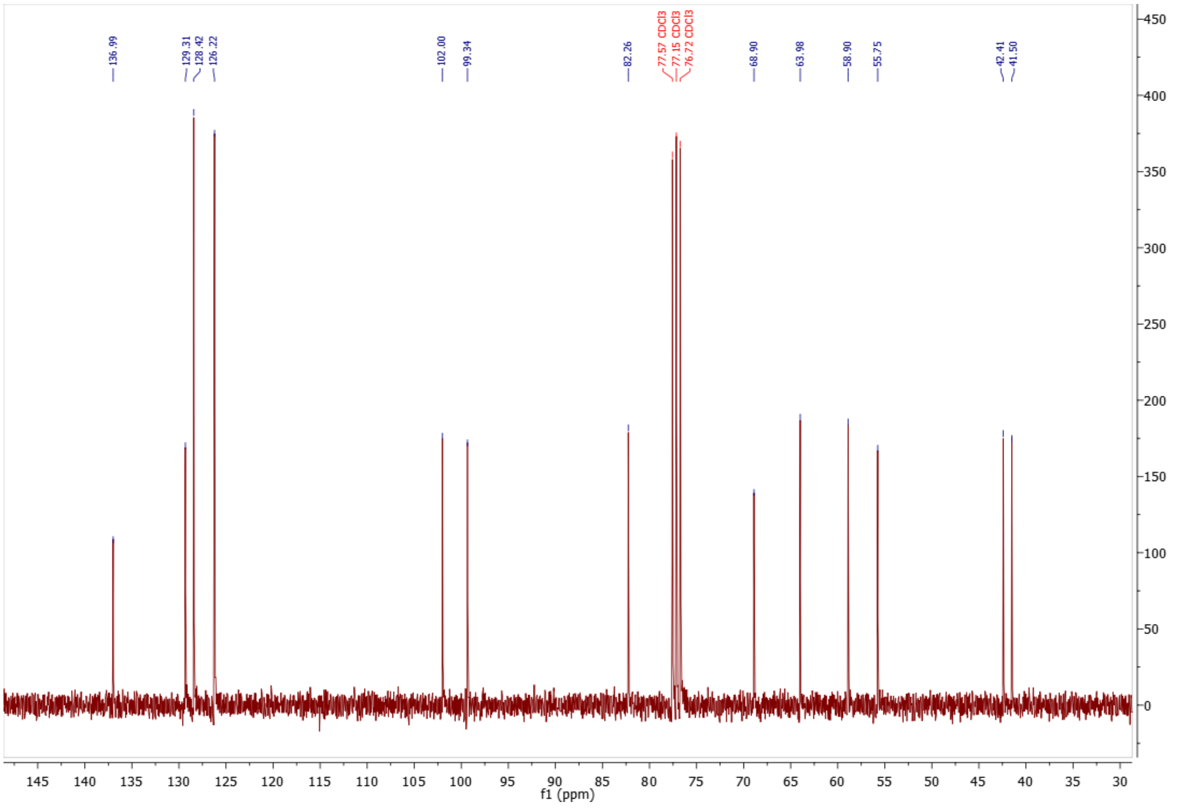

Fig. S21. ^{1}H NMR and ^{13}C NMR Spectrum of Compound **27**

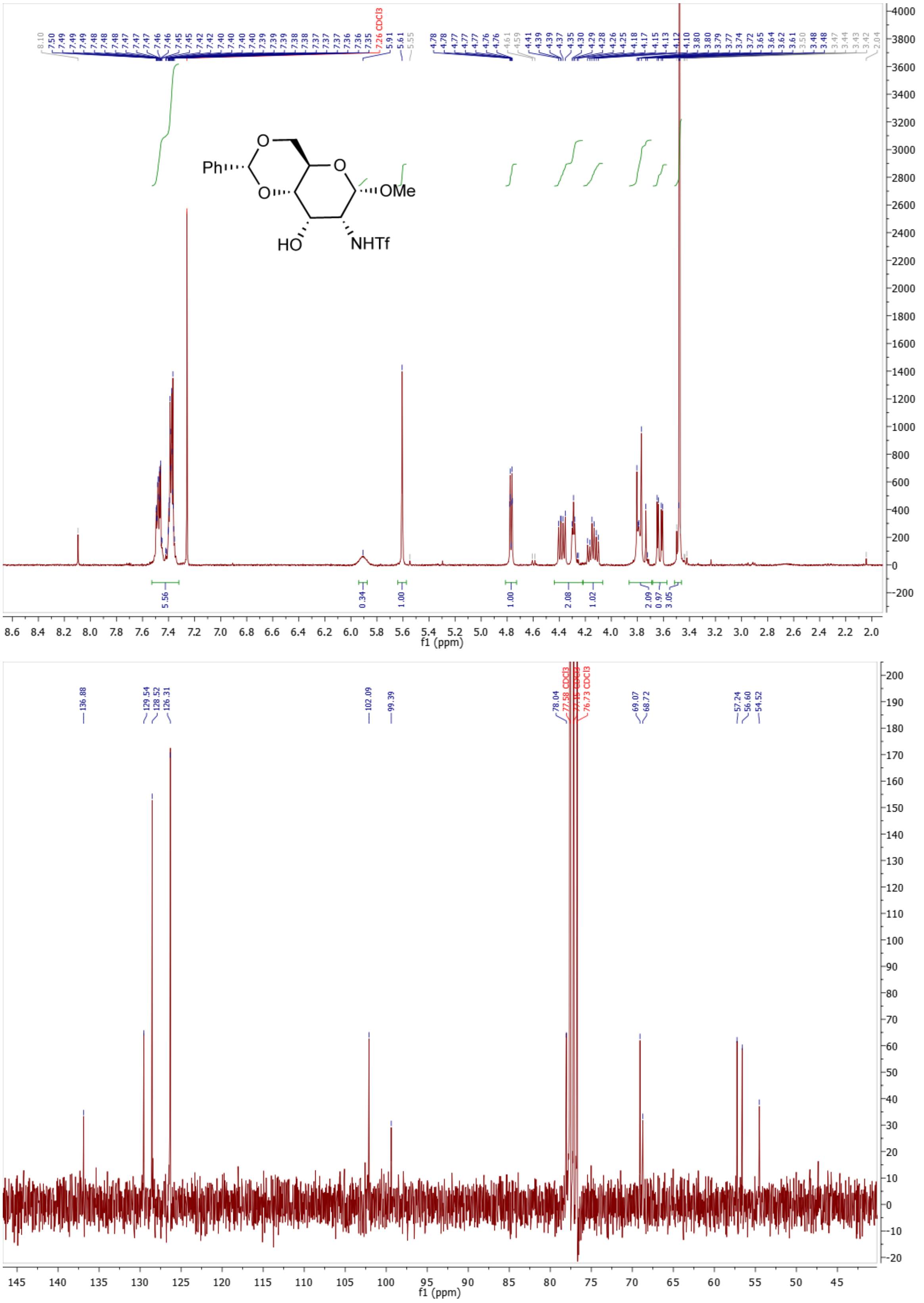

Fig. S22. ^{1}H NMR and ^{13}C NMR Spectrum of Compound **28**

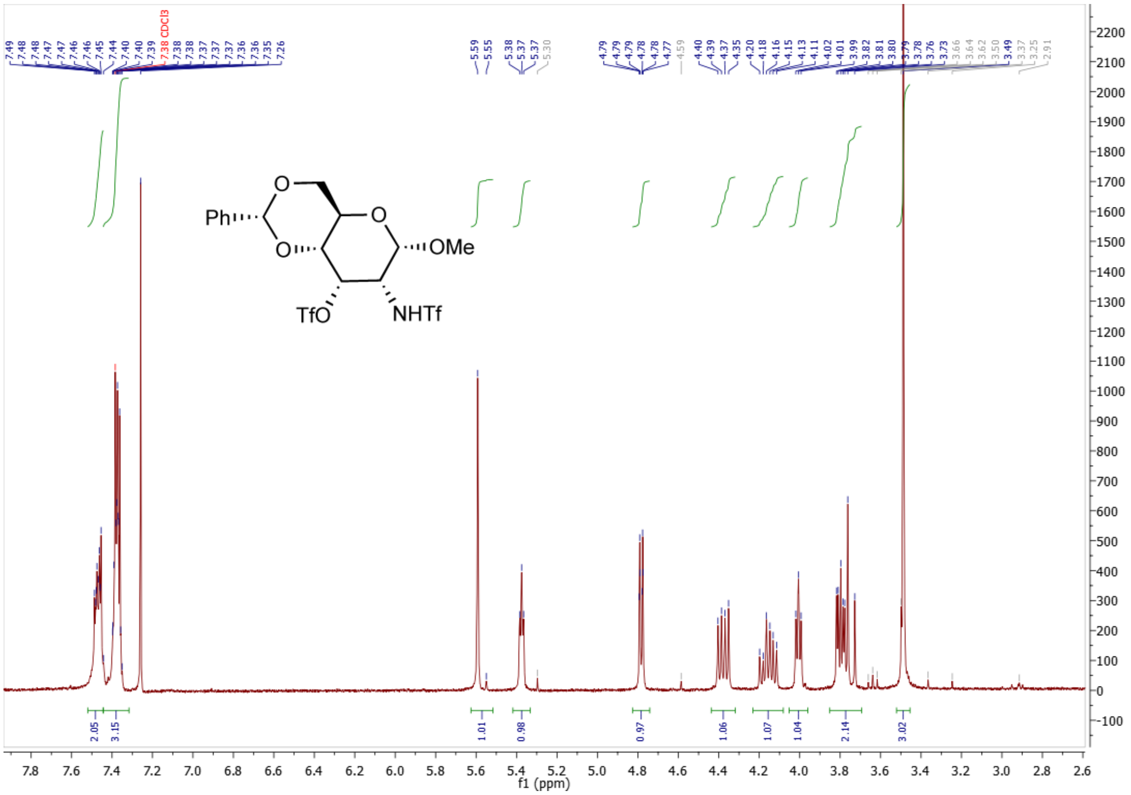

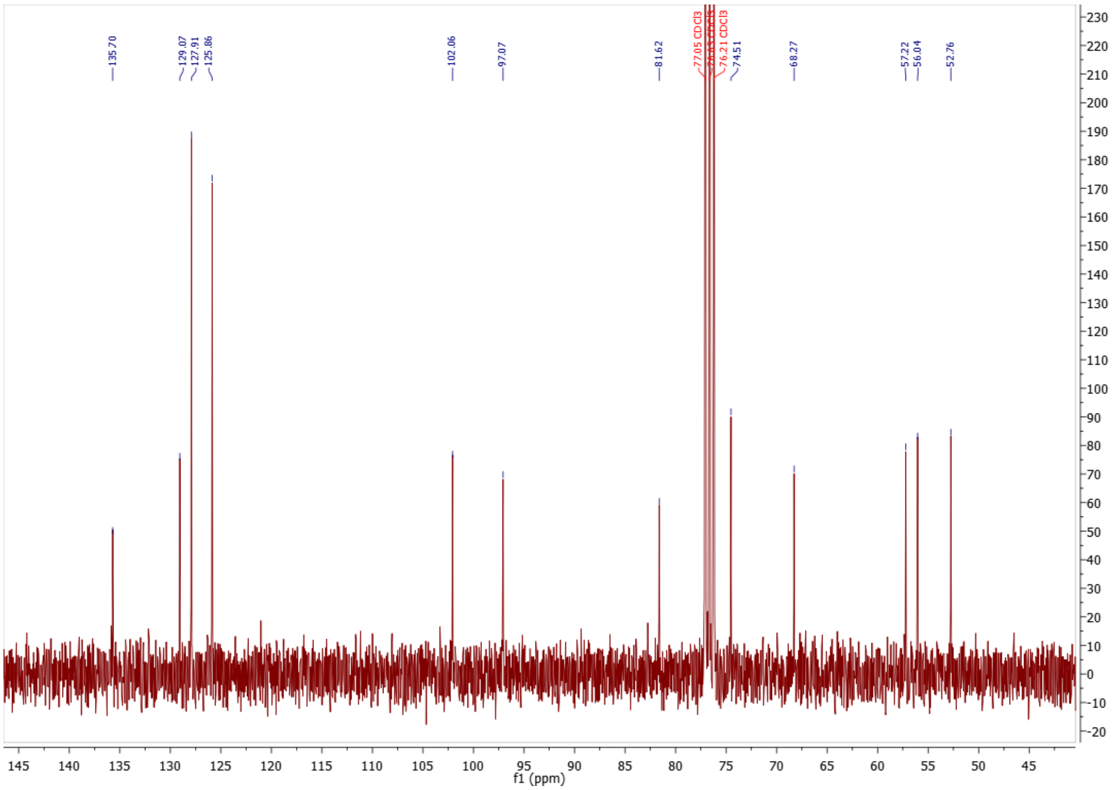

Fig. S23. ^{1}H NMR and ^{13}C NMR Spectrum of Compound **29**

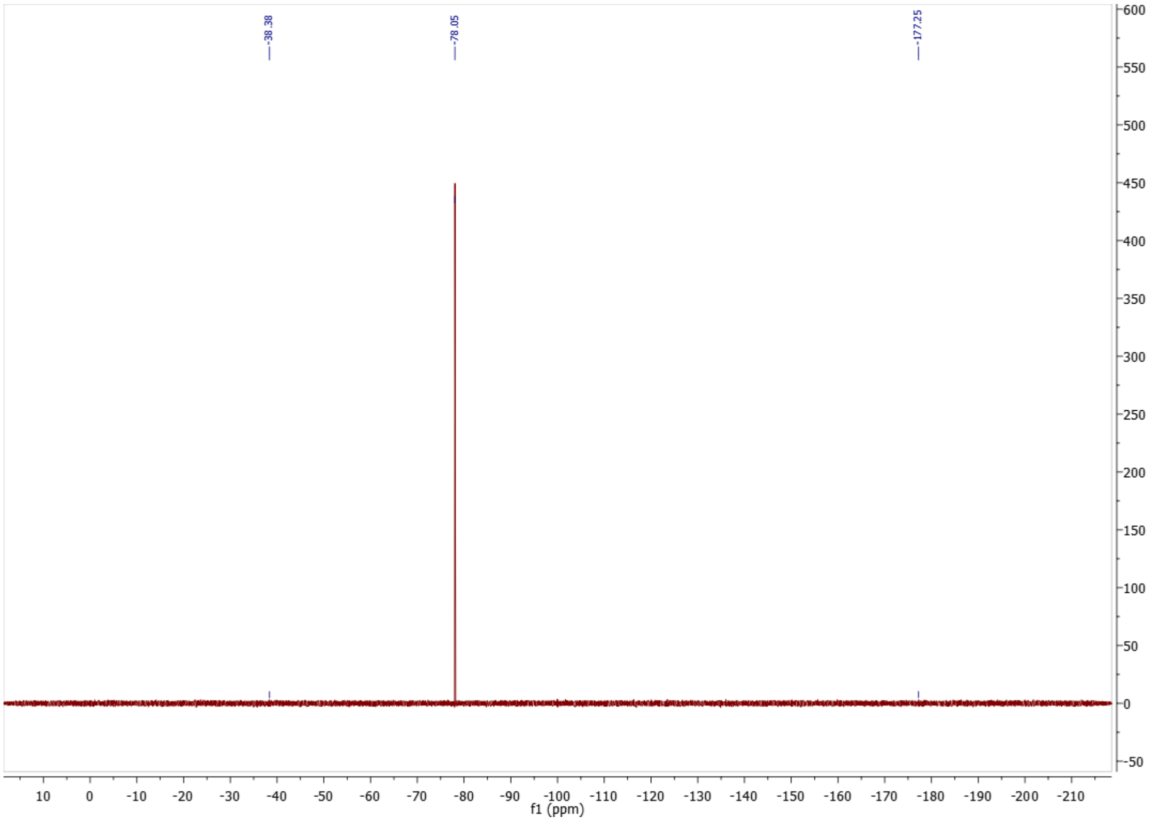

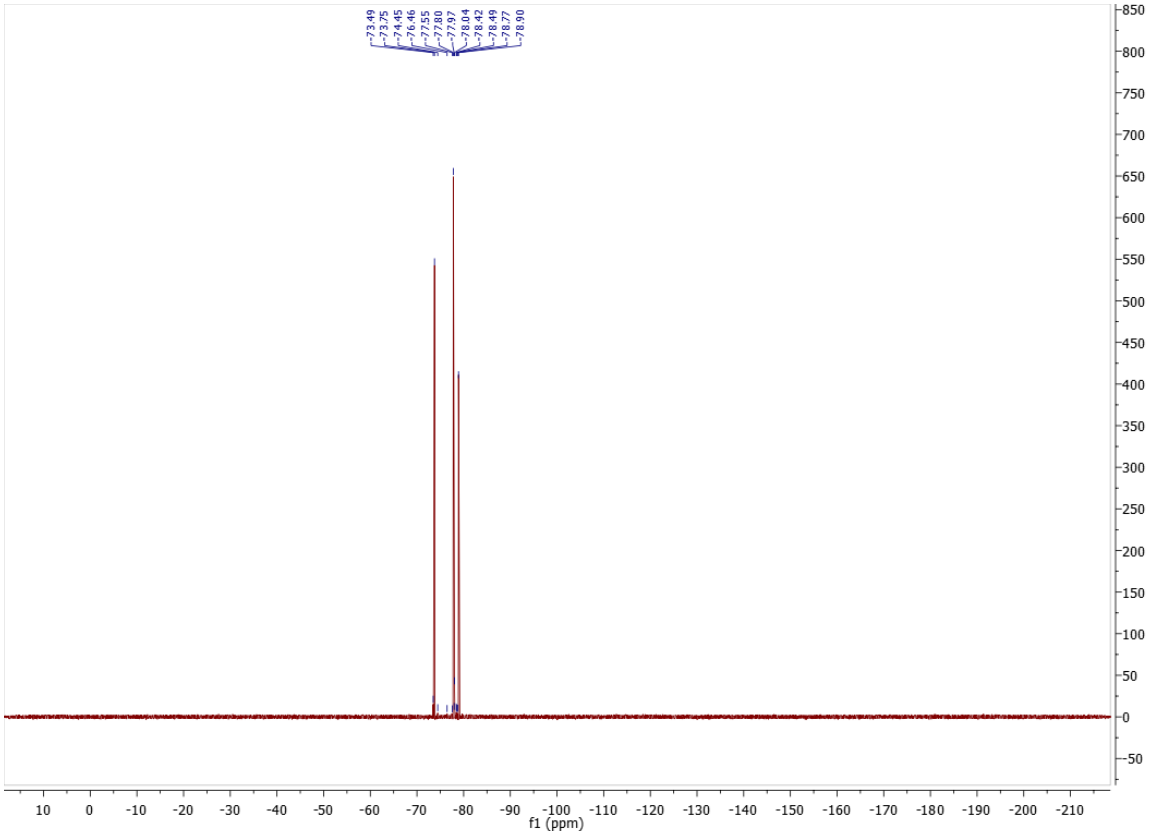

Fig. S24. ^{19}F NMR Spectrum of Compound **28 & 29**

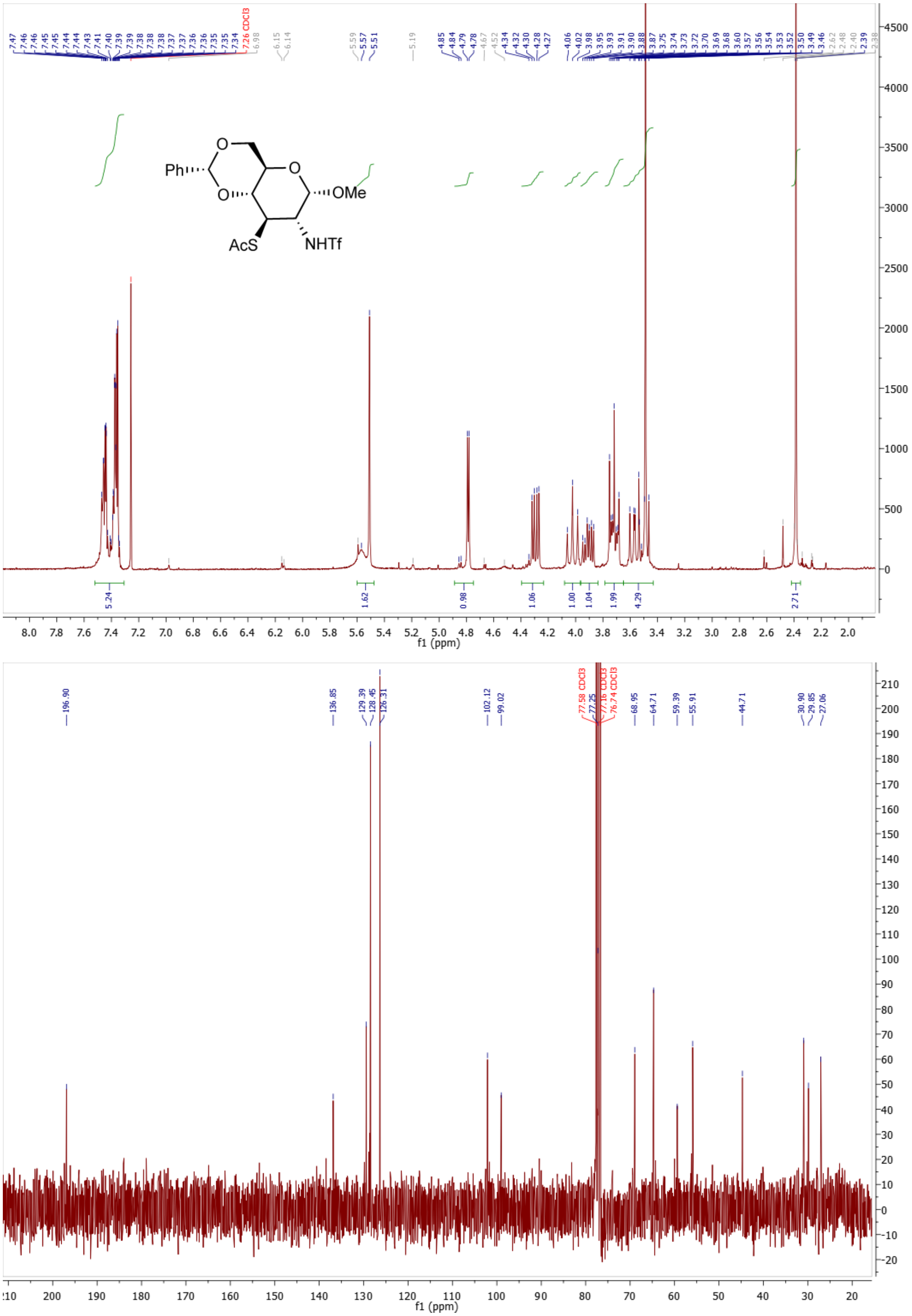

Fig. S25. ^{1}H NMR and ^{13}C NMR Spectrum of Compound **30**

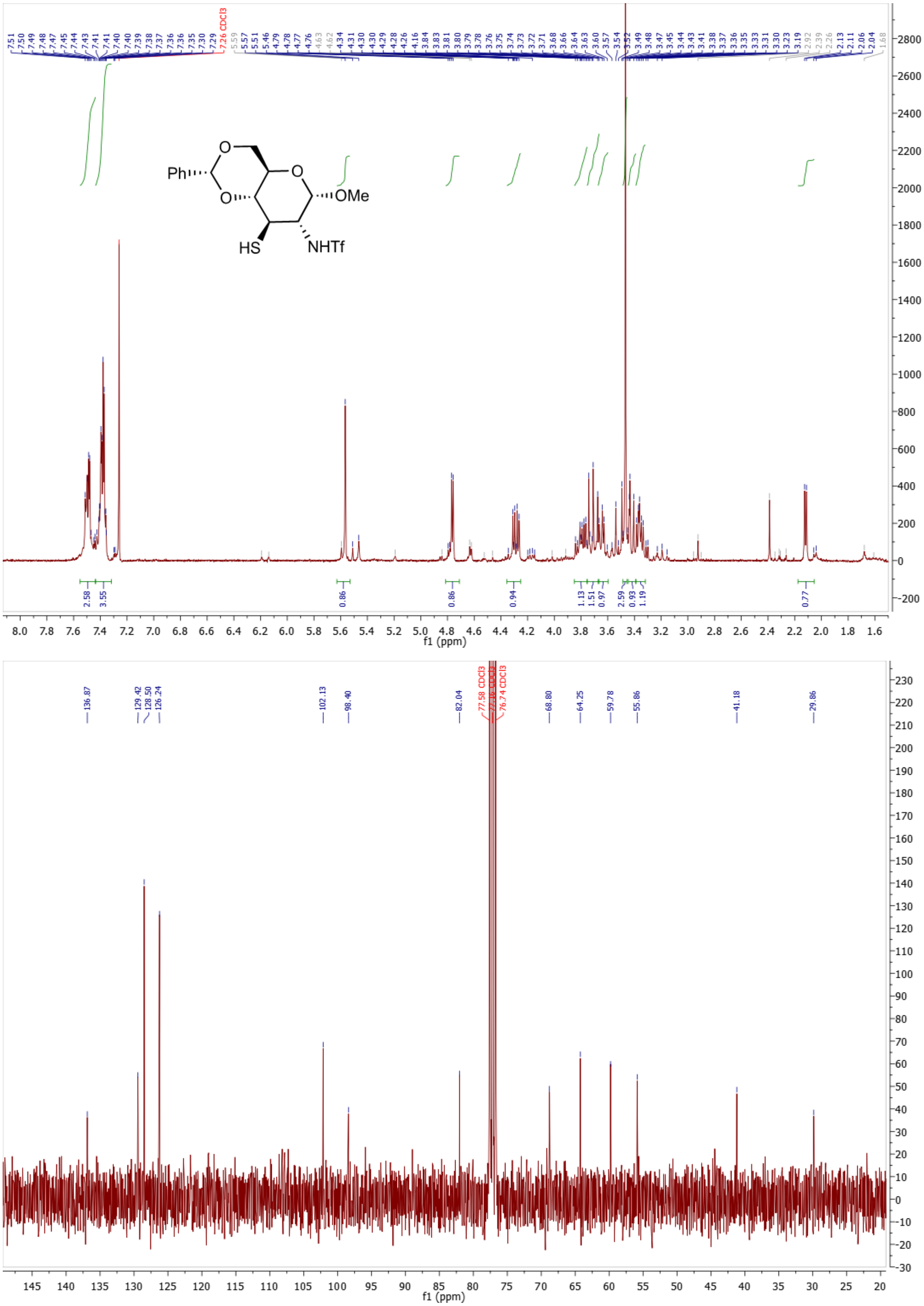

Fig. S26. ^{1}H NMR and ^{13}C NMR Spectrum of Compound **31**

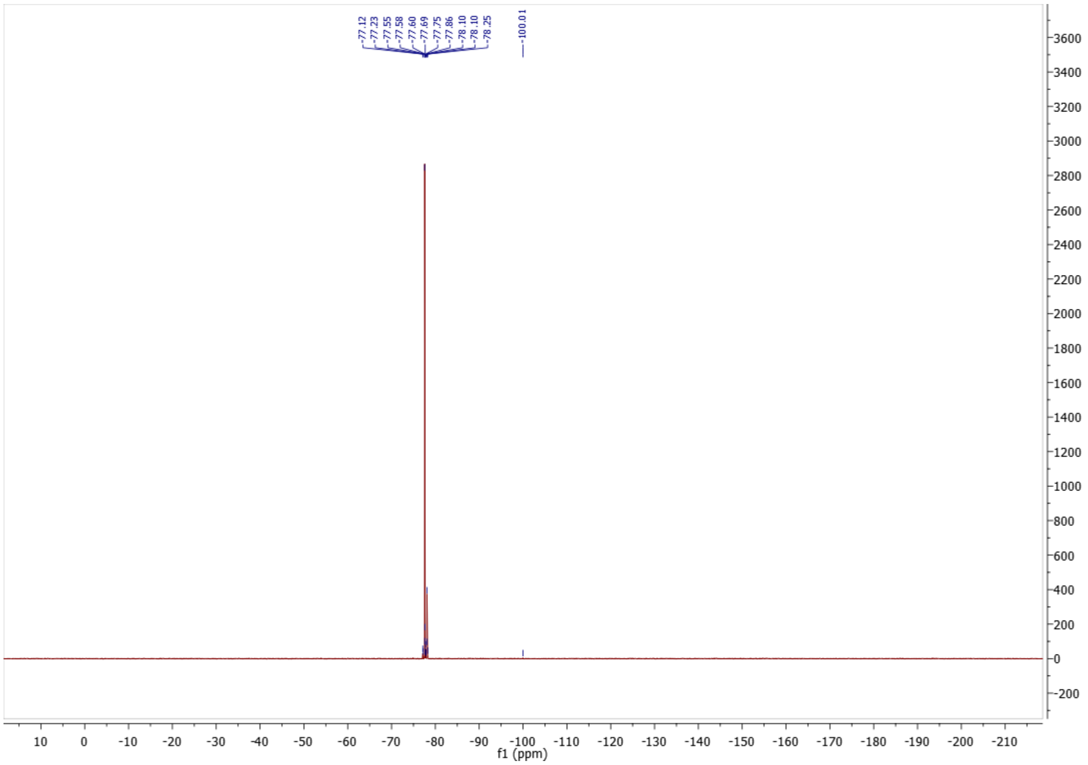

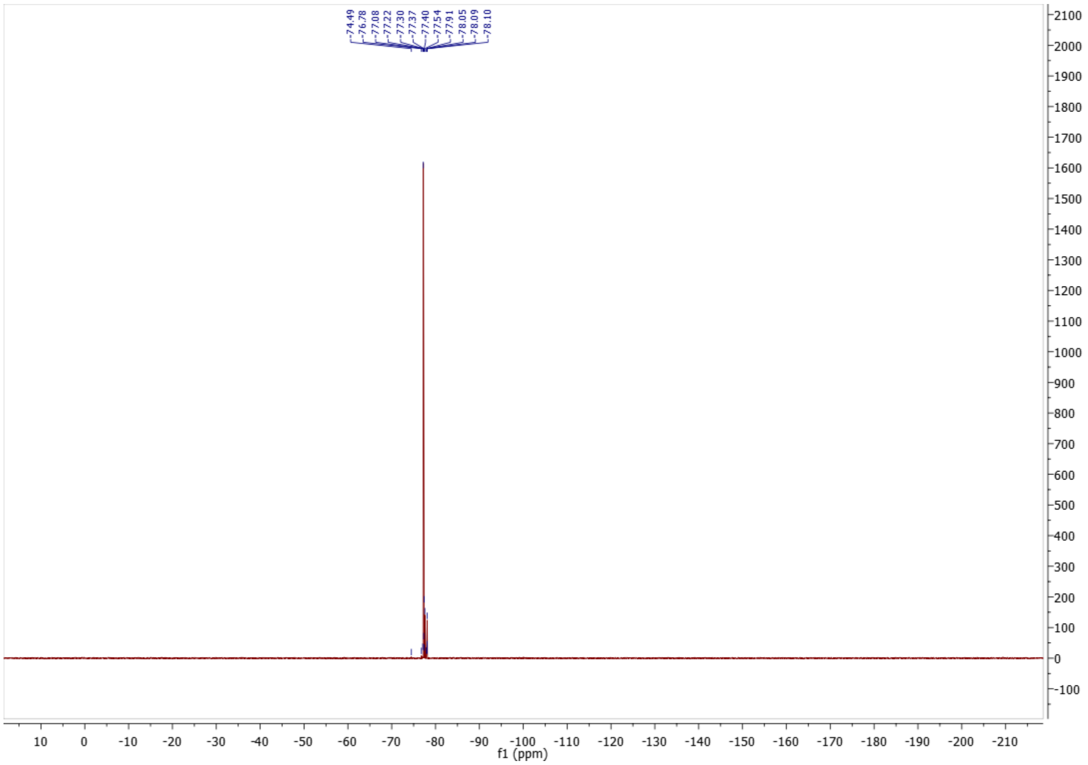

Fig. S27. ^{19}F NMR Spectrum of Compound **30 & 31**
